# Identification of TNFAIP3 as relapse biomarker and potential therapeutic target for MOG antibody associated diseases

**DOI:** 10.1038/s41598-020-69182-w

**Published:** 2020-07-24

**Authors:** Shrishti Saxena, Hrishikesh Lokhande, Grace Gombolay, Radhika Raheja, Timothy Rooney, Tanuja Chitnis

**Affiliations:** 1 Ann Romney Center for Neurologic Disease, Brigham and Women’s Hospital, Harvard Medical School, 60 Fenwood Road, Office 9002K, Boston, MA 02115-6128 USA; 2 Emory University and Children’s Healthcare of Atlanta, Atlanta, GA 30329 USA; 3 Department of Neurology, Partners Pediatric Multiple Sclerosis Center, Massachusetts General Hospital, Boston, MA USA

**Keywords:** Biomarkers, Neurology

## Abstract

MOG-antibody associated disease (MOG-AAD) is a recently recognized demyelinating disorder predominantly affecting children but also occurs in adults, with a relapsing course in approximately 50% of patients. We evaluated peripheral blood mononuclear cells from MOG-AAD patients by flow cytometry and found a strong antigen specific central memory cell (CMC) response with increased Th1 and Th17 cells at the time of a relapse. Transcriptomic analysis of CMCs by three independent sequencing platforms revealed TNFAIP3 as a relapse biomarker, whose expression was down regulated at a relapse compared to remission in MOG-AAD patients. Serum in an additional cohort of patients showed decreased TNFAIP3 levels at relapse compared to remission state in MOG-AAD patients. Our studies suggest that alterations in TNFAIP3 levels are associated with relapses in MOG-AAD patients, which may have clinical utility as a disease course biomarker and therapeutic target.

## Introduction

Myelin oligodendrocyte glycoprotein antibody associated diseases (MOG-AAD) have been recently described in approximately 25% of children and 5% of adults with demyelinating disorders^1,2^. MOG is a component of myelin expressed in the central nervous system (CNS), and MOG antibodies can be detected by cell-based assays in the serum^3,4^. MOG-AAD exhibits an age-related array of clinical phenotypes ranging from acute disseminated encephalomyelitis (ADEM), clinically isolated syndrome (CIS), optic neuritis (ON), recurrent forms of ADEM and ON, transverse myelitis (TM) and neuromyelitis spectrum disorder (NMO-SD)^2,3,5,6^. Approximately 50% of patients experience a multiphasic disease course^5,7,8^. There is an increasing body of literature demonstrating that patients with MOG-AAD have distinct clinical and MRI features from multiple sclerosis (MS)^9–11.^ Treatment response varies between MOG-AAD patients, and immunosuppressive treatments including rituximab or mycophenolate mofetil often result in incomplete control of disease^12^. Moreover, standard multiple sclerosis medications such as beta interferon may exacerbate disease^13,14^.

Individual patients with MOG-antibodies experience varying disease courses, some with frequent relapses, which can result in significant disability^13^. One of the main challenges in clinical care is to identify patients who will develop a multiphasic disease course, and require chronic immunotherapy. The persistent presence of high titers of serum MOG antibody titers has been studied in the prognostication of a multiphasic disease and relapses, although with inconsistent results^15,16^. Patients with MOG-antibodies have also been shown to have a higher frequency of MOG-reactive T cells suggesting an antigen-specific response, which may play a key role in disease initiation^17^, however have not been studied as disease biomarkers. In classic autoimmune disease, antigen-presenting cells, such as dendritic cells present a specific antigen to T cells to initiate an immune response. In the case of diseases characterized by self-antigen specific antibodies, it is likely that plasma cells and B cells are involved and cooperate with T cells to elicit autoimmunity^18^. In this study, we evaluate serological and T cell transcriptomic profiling in blood samples from patients with MOG-AAD at the time of a relapse versus remission with the goal of identifying mechanisms and biomarkers of relapse in this disease.

## Results

### Flow cytometric analysis of MOG-reactive CMC T cells show an increased percentage of IL17+, IFNγ+ and IL17+/IFNγ+ cells

**Figure 1:**
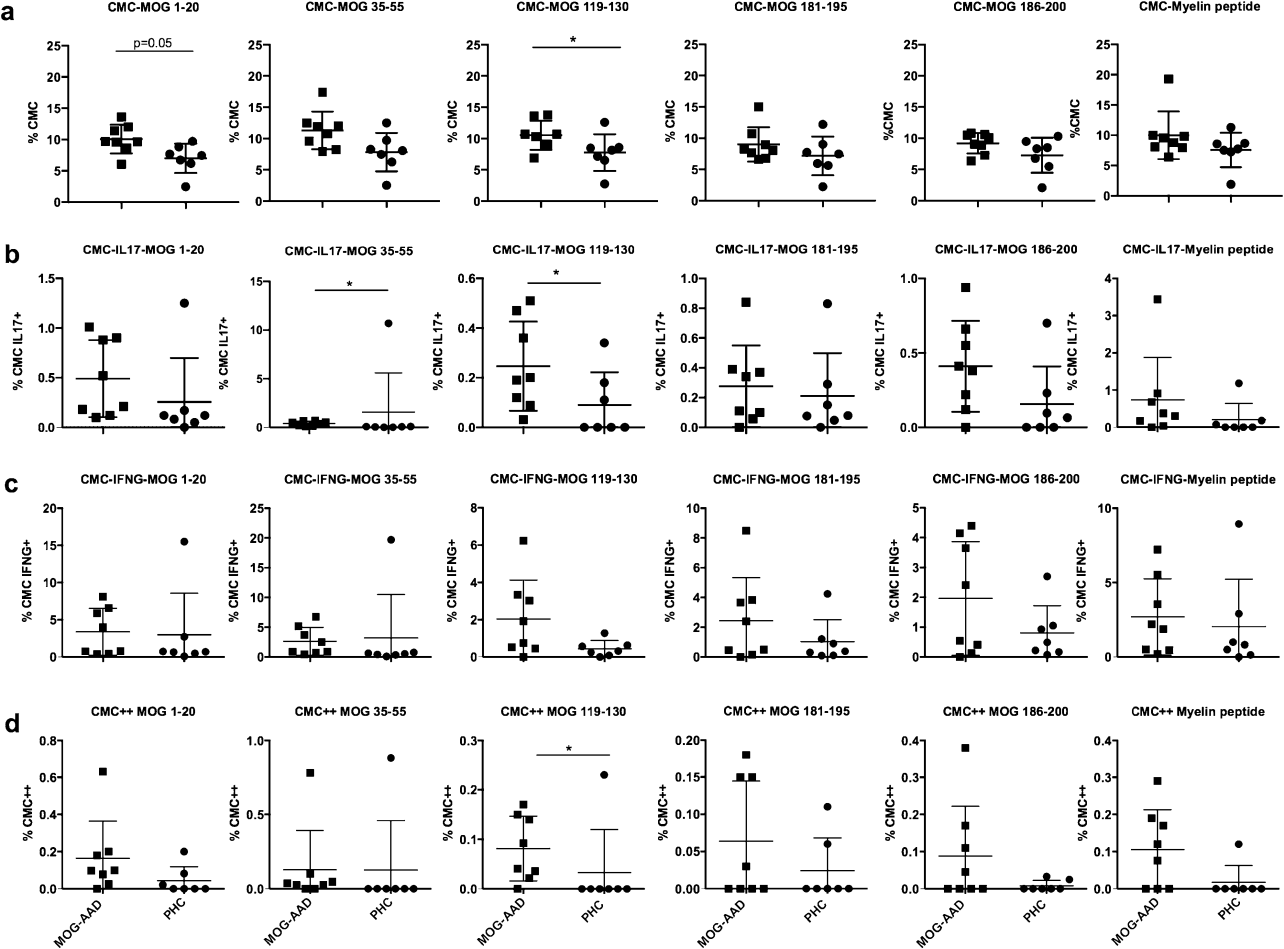
Cytokine expression in MOG-reactive central memory cells (CMCs) from untreated MOG-AAD patients and pediatric healthy controls (PHCs): PBMCs were stimulated and cultured with 10 μg/ml of MOG peptides (p1-20, p35-55, p119-130, p181-195 and p186-200) and cocktail of myelin peptides (Myelin Phospholipid Protein 139–154 (PLP), Myelin Basic Protein (MBP) 13–32, MBP 111–129 and MBP 146–170) for 7 days followed by staining with antibodies and flow cytometry as described in Materials and Methods. Data was analyzed using Flowjo vX.0.7 and the graphs were made using GraphPadPrism version 8.4.2 (464) **A** Percentage of CMCs (CD4+CCR7+CD45RA−). **B** Percentage of IL17+producing CMCs (CD4+CCR7+CD45RA-, IL17+). **C** Percentage of IFNγ+producing CMCs (CD4+CCR7+CD45RA−,IFNγ +). **D** Percentage of IL17+ and IFNγ+ producing CMCs (CD4+CCR7+CD45RA−,IL17+,IFNγ+). MOG-AAD n = 8, PHC n = 7, Mann–Whitney test, *P < 0.05.

Prior studies have demonstrated an increased T cell response to MOG peptides in MOG-AAD patients compared to controls.^17^ Here, we sought to evaluate the percentage of IL17+ and IFNγ+ cells in CMCs of untreated MOG-AAD patients (n = 8) and age and sex matched PHCs (n = 7) (Table 1 and Supplementary Table 1a), when stimulated with peptides MOG p1-20, MOG p35-55, MOG p119-130, MOG p181-195, MOG p186-200 and myelin peptides. We found an increase in the percentage of CMCs, as well as IL17+ and IL17+/IFNγ+ (double positive) CMCs in MOG-AAD patients when compared to PHCs (Fig. 1). (CMC-MOG 1–20, *P* = 0.05, CMC-MOG 119–130, *P* = 0.03; CMC-IL17+-MOG 35–55, *P* = 0.02, CMC-IL17+-MOG 119–130, *P* = 0.03; CMC++MOG 119–130, *P* = 0.04).

**Table 1:** Demographics of MOG-AAD subjects and controls studied.

Subjects	Number	Age range (years)	Sex ratio F:M
MOG-antibody associated disease	24	4.5–52	15:9
Pediatric healthy controls	44	4–18	26:18

**Figure 2:**
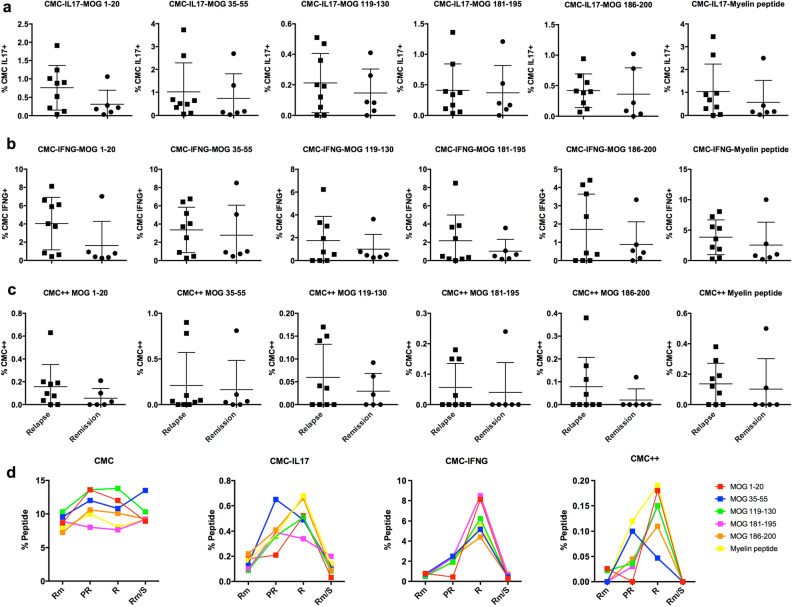
Cytokine expression in MOG-reactive central memory cells (CMCs) from MOG-AAD patients at the time of relapse and remission: PBMCs were stimulated and cultured with 10 μg/ml of MOG peptides (p1-20, p35-55, p119-130, p181-195 and p186-200) and cocktail of myelin peptides (Myelin Phospholipid Protein 139–154, Myelin Basic Protein (MBP) 13–32, MBP 111–129 and MBP 146–170) for 7 days followed by staining with antibodies and flow cytometry as described in Materials and Methods. Data was analyzed using Flowjo vX.0.7 and the graphs were made using GraphPadPrism version 8.4.2 (464) **A** Percentage of IL17+producing CMCs (CD4+CCR7+CD45RA−, IL17+). **B** Percentage of IFNγ+producing CMCs (CD4+CCR7+CD45RA−,IFNγ+). **C** Percentage of IL17+ and IFNγ+ producing CMCs (CD4+CCR7+CD45RA−,IL17+,IFNγ+). Relapse n = 9, Remission n = 6 **D** CMCs (CD4+CCR7+CD45RA–), CMC IL17+ (CD4+CCR7+CD45RA−,IL17 +), CMC IFNγ+ (CD4+CCR7+CD45RA−, IFNγ+), CMC++ (CD4+CCR7+CD45RA−,IL17+, IFNγ+) analysis from a single pediatric MOG-AAD patient#1 with longitudinal samples, (1) Rm (remission, MOG-AAD#1.2), (2) PR (pre-relapse, MOG-AAD#1.3), (3) R (relapse, MOG-AAD#1.4) and (4) Rm/S (remission treated with steroid, MOG-AAD#1.5).

We next identified a group of MOG-AAD patients with a blood sample within 30 days prior to a relapse (n = 9) and during a remission time point (non-relapse, n = 6) (Supplementary Table 1a). Interestingly, we found an increased proportion of IL17+, IFNγ+ and IL17+IFNγ+ (double positive) CMCs after stimulation with several individual MOG peptides in MOG-AAD patients at the time of relapse as compared to remission time point (Fig. 2a–c).

Furthermore, PBMCs from MOG-AAD patient#1 with 4 longitudinal samples, (1) remission (MOG-AAD#1.2) 2) pre-relapse (MOG-AAD#1.3) time point which was 2 months prior to a relapse during which the patient experienced headache and mild visual symptoms, (3) relapse (MOG-AAD#1.4) characterized by bilateral weakness and multiple new MRI lesions and (4) remission (MOG-AAD#1.5) on treatment with steroid were stimulated with individual MOG peptides. We found a dramatic increase in CMC IL17+ and CMC IFNγ+cells at a relapse. Specifically, there was an increase in CMC IL17+cells responsive to MOG p35-55 in the 2 months prior to fulminant relapse, when the patient had mild visual symptoms (pre-relapse state) (Fig. 2d).

### Gene expression analysis revealed and confirmed a relapse biomarker in MOG-AAD patients

Since our initial results demonstrated that CMC T cells might play a role in relapse and in multiphasic disease course in MOG-AAD patients, we sought to further evaluate the transcriptomic profile of these cells. Single cell RNA sequencing (inDrop) was performed on an untreated MOG-AAD patient#1 with 3 longitudinal samples during remission (MOG-AAD#1.2), pre-relapse (MOG-AAD#1.3) and at a relapse (MOG-AAD#1.4). The relapse sample failed sequencing, however the pre-relapse and remission samples were evaluable. Given the evolving understanding that in MOG-AAD, clinical symptoms can occur prior to the presence of new MRI lesions^19^, moving forward we re-classified the pre-relapse sample as relapse. Analysis was performed as described in “Materials and methods”.

**Figure 3:**
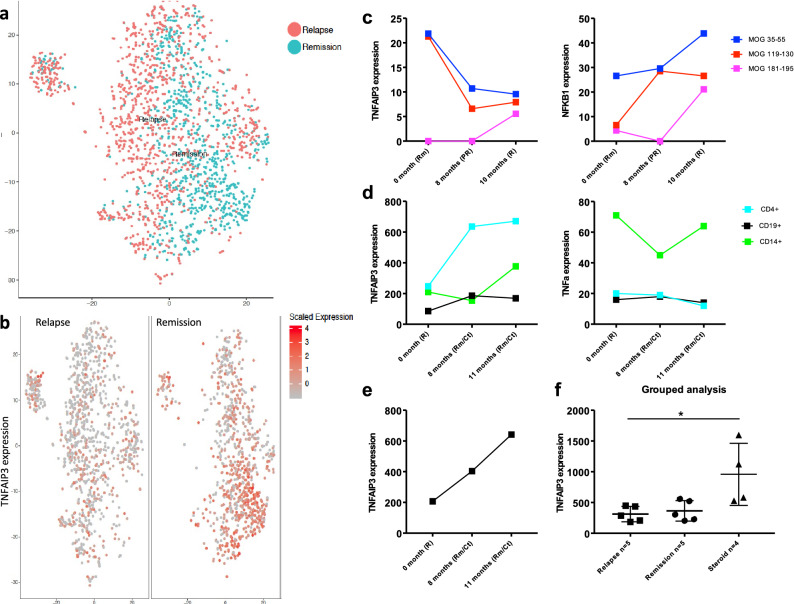
Gene expression analysis in PBMCs from MOG-AAD patients: Single cell RNA sequencing (inDrop) was performed on an untreated MOG-AAD patient#1 with 2 longitudinal samples, (1) Remission (MOG-AAD#1.2) and (2) Pre-relapse (MOG-AAD#1.3) as described in Materials and Methods. cDNA libraries were sequenced using the Illumina NextSeq 500 platform and analyzed following V3 Indrop criteria. After sequencing the raw BCL files were demultiplexed using bcl2fastq software by illumina (https://support.illumina.com/sequencing/sequencing_software/bcl2fastq-conversion-software.html). Reads obtained from bcl2fastq were further processed using the single-cell RNA-seq pipeline of the bcbio-nextgen (https://bcbio-nextgen.readthedocs.io/en/latest/contents/pipelines.html#single-cell-rna-seq) software suite. The scaled data was further clustered using Seurat and visualized using TSNE (https://www.biorxiv.org/content/early/2018/11/02/460147). **A** Cluster analysis of a relapse and remission sample. **B** Differential expression of TNFAIP3 in the relapse and remission sample by single cell sequencing. Digital Gene Expression (DGE) sequencing was performed on an untreated MOG-AAD patient#1 with 3 longitudinal samples, (1) Rm (remission, MOG-AA#1.2), (2) PR (pre-relapse, MOG-AAD#1.3), and (3) R (relapse, MOG-AAD#1.4) as described in Materials and Methods. Raw BCL files generated through sequencing were further de-multiplexed using Picard (https://github.com/broadinstitute/picard) and the resulting FASTQ files where aligned to the human reference genome (GRCh38) using the STAR v2.4.2a^52^ aligner. Further QC was done using the RNA-seQC^53^ and transcript counts were produced using feature Counts function of the Subread package^54^. Data was normalized using the DESeq2 package^55^ and the graphs were made using GraphPadPrism version 8.4.2 (464). **C** TNFAIP3 and NFκβ1 expression by DGE sequencing. NanoString Gene Expression Assay was performed on a MOG-AAD patient#2 with 3 longitudinal samples, (1) untreated R (relapse, MOG-AAD#2.1), (2) 8 months Rm/Ct (mycophenolate mofetil treated at remission, MOG-AAD#2.2), and (3) 11 months Rm/Ct (mycophenolate mofetil treated at remission, MOG-AAD#2.3) as described in Materials and Methods. Data were normalized and analyzed using nSolver software via the geometric mean of included housekeeping genes. The graphs were made using GraphPadPrism version 8.4.2 (464). **D** TNFAIP3 and TNF-α expression by NanoString Gene Expression Assay. qPCR was performed on CD4+ T cells from 7 MOG-AAD patients with longitudinal samples as described in Materials and Methods. It also included MOG-AAD patient#2 with 3 longitudinal samples as previously used for NanoString gene expression assay. The graphs were made using GraphPadPrism version 8.4.2 (464). **E** TNFAIP3 expression in MOG-AAD patient#2 by qPCR. **F** Grouped analysis of TNFAIP3 expression in relapse samples, remission samples and samples treated with corticosteroids by qPCR. Relapse n = 5, Remission n = 5, Steroid n = 4, Ordinary 1-way ANOVA; *P* = 0.0137.

Cluster analysis of all the cells from relapse and remission samples using Seurat indicated that gene expression was homogeneous and there were no obvious differences between cells of relapse and remission samples (Fig. 3a). However, to identify genes that were differentially expressed in relapse and remission samples, we calculated average gene expression between the relapse and remission samples and subtracted gene expression values from relapse and remission samples for each gene. From this analysis, we chose the top 5 differentially expressed genes that were up regulated in the relapse and remission samples. We demonstrated differential expression of TNFAIP3 (Tumor Necrosis Factor Inducible Protein A20), which was higher in the remission sample as compared to relapse sample of a MOG-AAD patient (Fig. 3b). The other top genes identified to be up regulated during remission were HILPDA (Hypoxia Inducible Lipid Droplet-Associated), MXI-1 (MAX Interactor 1, Dimerization Protein), BNIP3 (BCL2 Interacting Protein 3), FAM162A (Family With Sequence Similarity 162 Member A); while genes up regulated during relapse included SNRPG (Small Nuclear Ribonucleoprotein Polypeptide G), EWSR1 (Ewing Sarcoma RNA Binding Protein 1), AL137058.2 (Clone-based Ensemble gene), HMGN1 (High Mobility Group Nucleosome Binding Domain 1), LRRC75A-AS1/ SNHG29 (Small Nucleolar RNA Host Gene 29) (Supplementary Fig. 1).

In order to validate our single cell RNA sequencing findings, we sought to evaluate the same MOG-AAD patient#1 with 3 longitudinal samples during remission (MOG-AAD#1.2), pre-relapse (MOG-AAD#1.3) and at a relapse (MOG-AAD#1.4) using DGE sequencing. DGE sequencing is a cost-effective method that offers low background noise, increased sensitivity and reproducibility^24^. For this, PBMCs were stimulated with individual MOG peptides, p35-55, p119-130 and p181-195. Total RNA from CMC T cells was isolated and sequenced. Broad Genomics Platform sequenced the libraries. Consistent with the single cell RNA sequencing findings, we found that gene expression of TNFAIP3 was decreased at both the pre-relapse and relapse sample compared to the remission sample. TNFAIP3 is known to be a regulator of NFκβ. We found that the gene expression of NFκβ1 was increased at relapse compared to remission time point, the inverse of TNFAIP3 expression (Fig. 3c). The other genes identified by single cell RNA sequencing, namely HILPDA, MXI-I, BNIP3 and FAM162A showed a similar trend by DGE sequencing. They were up regulated during remission as compared to pre-relapse and relapse time points (Supplementary Fig. 2a). However, SNRPG, EWSR1, HMGN1 and LRRC75A-AS1, that were shown to be up regulated during pre-relapse time point by single cell RNA sequencing did not follow the same trend by DGE sequencing (Supplementary Fig. 2b). Gene expression for AL137058.2 was below the detection limit by DGE sequencing.

To further confirm our RNA sequencing results, we decided to test another MOG-AAD patient#2 with 3 longitudinal samples, 1 at relapse (MOG-AAD#2.1) and 2 mycophenolate mofetil treated samples at remission (MOG-AAD#2.2 and MOG-AAD#2.3), using NanoString’s differential gene expression platform. NanoString is a high throughput technique that allows simultaneous gene expression of more than 700 genes. We analyzed gene expression in CD4+ T cells, CD19+ B cells and CD14+monocytes using the nCounter software. There was a distinct increase in the TNFAIP3 expression from CD4+ T cells in remission samples as compared to relapse sample thereby indicating that CD4+ T cells play an important role in TNFAIP3 regulation (Fig. 3d). In contrast, TNF-α expression from CD14+ monocytes, principal source of TNF-α in humans was increased in the relapse sample as compared to remission. CD4+ T cells followed a similar trend, where in TNF-α expression was higher at relapse as compared to remission samples.

To validate NanoString gene expression assay results, we isolated CD4+ T cells from 7 additional MOG-AAD patients with longitudinal samples (longitudinal samples n = 5/7, relapse n = 7 and remission n = 8, total n = 15). It also included MOG-AAD patient#2 with 3 longitudinal samples as previously used for NanoString gene expression assay. Quantitative real-time polymerase chain reaction (qPCR) was performed using FAM-labeled primer for TNFAIP3. GAPDH gene was used as an endogenous control to normalize for differences in the amount of total RNA in each sample. All values are shown as relative expression. The qPCR data replicated the results from NanoString gene expression assay. There was an increase in the relative expression of TNFAIP3 at remission time points and a decrease at relapse in the CD4+ T cells of MOG-AAD patient#2 (Fig. 3e). We next conducted grouped analysis of CD4+ TNFAIP3 expression levels from patients in relapse or remission states on disease modifying therapies, and samples from patients receiving high dose of corticosteroids, which are known to induce TNFAIP3 through binding of the glucocorticoid receptor^20^. Grouped analysis comparing relapse samples (n = 5), remission samples (n = 5) and samples from patients treated with high dose of corticosteroids (n = 4) showed a significant difference between the three groups (Fig. 3f) (Ordinary 1-way ANOVA; *P* = 0.013).

### Protein expression analysis demonstrated decreased TNFAIP3 expression in MOG-AAD patient at relapse timepoint

**Figure 4:**
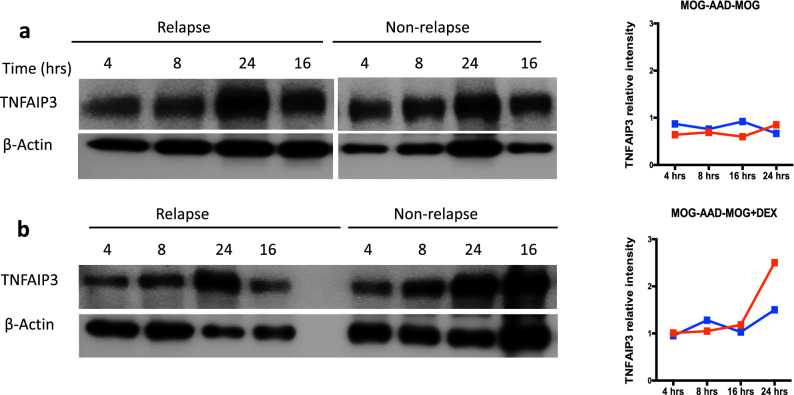
TNFAIP3 protein expression in relapse and non-relapse samples from an untreated MOG-AAD patient: PBMCs from a MOG-AAD patient#3 at a relapse (MOG-AAD#3.1) and non-relapse (MOG-AAD#3.2) time point were cultured under different conditions, analyzed by SDS-PAGE and followed by western blot for TNFAIP3 at the indicated time points (hours) as described in Materials and Methods. **A** MOG antigen stimulation at 1 μg/ml (peptide cocktail comprising of MOG p1-20, p35-55, p119-130, p181-195 and p186-200). **B** MOG antigen + Dexamethasone stimulation at 1 μg + 100 nm. Representative Western blot images are shown on left and protein bands quantified using ImageJ version 1.53b and normalized to β-actin are shown on the right. The graphs were made using GraphPadPrism version 8.4.2 (464). Full-length blots/gels for TNFAIP3 and β-Actin are presented in Supplementary Fig. 5.

As the sequencing data on three independent platforms consistently suggested the differential expression of TNFAIP3 in MOG-AAD patients, we evaluated protein expression of TNFAIP3 in whole PBMCs from an untreated MOG-AAD patient#3 (relapse MOG-AAD#3.1 and non-relapse MOG-AAD#3.2). Ligation of the TCR has been shown to induce TNFAIP3 expression^21^ and corticosteroids are commonly used as treatment for MOG-AAD which can aid in the resolution of relapses and potentially prevent new relapses^5,7^. Hence, we cultured PBMCs with 2 conditions: MOG antigen stimulation (1 μg/ml) and MOG+dexamethasone stimulation (1 μg + 100 nm) at 4 timepoints: 4, 8, 16 and 24 h. Addition of MOG peptide did not increase TNFAIP3 expression in relapse or non-relapse samples (Fig. 4a, full-length blots/gels are presented in Supplementary Fig. 5a) but in the presence of both, MOG antigen and dexamethasone, which is a synthetic glucocorticoid, TNFAIP3 expression was comparable at 4, 8 and 16 h, however expression increased after 24 h of stimulation in the relapse sample as compared to non-relapse sample thereby partially rescuing the expression of TNFAIP3 in the relapse sample (Fig. 4b, full-length blots/gels are presented in Supplementary Fig. 5b).

We also studied a dose response at lower and higher concentration of MOG antigen and dexamethasone stimulations in a MOG-AAD patient#2 (untreated, relapse MOG-AAD#2.1 and treated with mycophenolate mofetil, non-relapse MOG-AAD#2.3). Here we cultured PBMCs with 5 conditions: exvivo (unstimulated), MOG antigen stimulation (lower dose at 1 μg/ml and higher dose at 10 μg/ml) and MOG + dexamethasone stimulation (lower dose at 1 μg + 100 nm and higher dose at 10 μg + 1000 nm) at 3 timepoints: 4, 16 and 24 h. TNFAIP3 expression increased in presence of dexamethasone as compared to MOG antigen alone stimulation, more so in the non-relapse MOG-AAD sample as compared to relapse and with higher dose of MOG + dexamethasone (Supplementary Fig. 3a–e, full-length blots/gels are presented in Supplementary Fig. 6). TNFAIP3 expression was also increased in the non-relapse MOG-AAD sample as compared to relapse MOG-AAD sample in the exvivo (unstimulated) condition (Supplementary Fig. 3f, full-length blots/gels are presented in Supplementary Fig. 6).

Since TNFAIP3 is a well-known regulator of NFκβ. We studied its correlation with NFκβ subunits p50 and p65 in the same MOG-AAD patient#2 at a lower dose of MOG antigen stimulation (1 μg/ml) at 4 timepoints: 4, 8, 16 and 24 h. There was a negative correlation of TNFAIP3 expression with NFκβ subunits p50 and p65 (Supplementary Fig. 4, full-length blots/gels are presented in Supplementary Fig. 7).

### Serum analysis showed decreased levels of TNFAIP3 at relapse and increased levels at remission in MOG-AAD patients and in healthy controls

**Figure 5:**
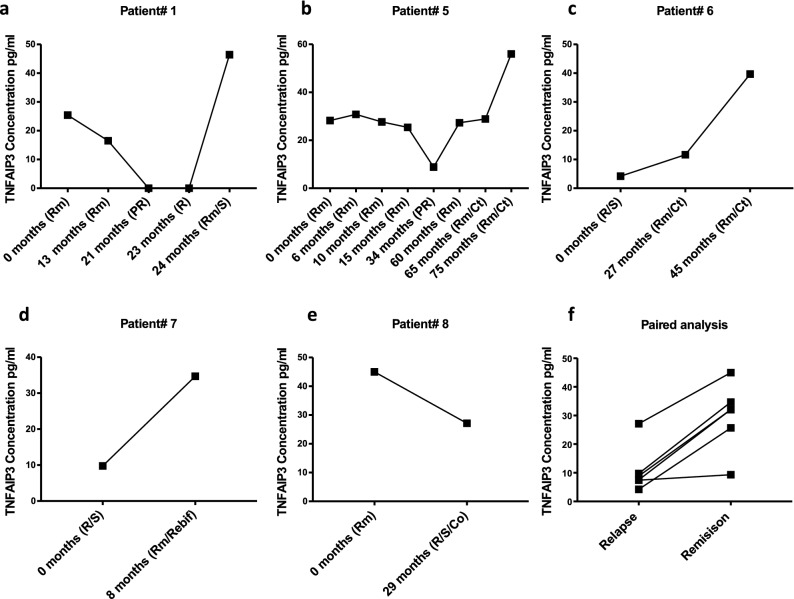
Serum levels of TNFAIP3 in indicated longitudinal samples of MOG-AAD patients determined by ELISA: The graphs were made using GraphPadPrism version 8.4.2 (464) **A** TNFAIP3 levels in MOG-AAD patient#1 with 5 longitudinal samples, (1) Rm (remission, MOG-AAD#1.1), (2) Rm (remission, MOG-AAD#1.2), (3) PR (pre-relapse, MOG-AAD#1.3), (4) R (relapse, MOG-AAD#1.4), and (5) Rm/S (remission treated with steroid, MOG-AAD#1.5)**. B** TNFAIP3 levels in MOG-AAD patient#5 with 8 longitudinal samples, (1) Rm (remission, MOG-AAD#5.1), (2) Rm (remission, MOG-AAD#5.2), (3) Rm (remission, MOG-AAD#5.3), (4) Rm (remission, MOG-AAD#5.4), (5) PR (pre-relapse, MOG-AAD#5.5), (6) Rm (remission, MOG-AAD#5.6), (7) Rm/Ct (mycophenolate mofetil treated at remission, MOG-AAD#5.7), and (8) Rm/Ct (mycophenolate mofetil treated at remission, MOG-AAD#5.8)**. C** TNFAIP3 levels in MOG-AAD patient#6 with 3 longitudinal samples, (1) R/S (relapse treated with steroid, MOG-AAD#6.1), (2) Rm/Ct (mycophenolate mofetil treated at remission, MOG-AAD#6.2), and (3) Rm/Ct (mycophenolate mofetil treated at remission, MOG-AAD#6.3)**. D** TNFAIP3 levels in MOG-AAD patient#7 with 2 longitudinal samples, (1) R/S (relapse treated with steroid, MOG-AAD#7.1), and (2) Rm/Rebif (remission treated with interferon beta-1a, MOG-AAD#7.2)**. E** TNFAIP3 levels in MOG-AAD patient#8 with 2 longitudinal samples, (1) Rm (remission, MOG-AAD#8.1) and (2) R/S/Co (remission treated with steroid and Glatiramer acetate, MOG-AAD#8.2)**. F** Mixed model paired analysis compared MOG-AAD patients, who had, both, relapse and remission samples. Relapse n = 8, Remission n = 22. The comparison between the relapse and remission group was done using Nonlinear Mixed-Effects Models library in R (nlme).

Previous studies measuring TNFAIP3 in the serum using ELISA assays have been performed in the context of viral infections such as chronic Hepatitis B infections^22^. To confirm that alterations in TNFAIP3 transcription corresponded with translation into the TNFAIP3 protein, we tested serum levels of TNFAIP3 in MOG-AAD patients. We found a strong correlation of TNFAIP3 serum level decrement with the onset of relapse and increase following intravenous steroids in MOG-AAD patient#1.1–1.5 (Fig. 5a). Serum from other MOG-AAD patients, #5–8 (Supplementary Table 1a), with longitudinal samples showed similar trends (Fig. 5b–e). Mixed model paired analysis between relapse (n = 8) and remission (n = 22) samples from MOG-AAD patients (6 pairs) showed a significant reduction in TNFAIP3 levels in paired relapse samples as compared to remission samples (*P* = 0.006) (Fig. 5f).

Further, using Nonlinear Mixed-Effects Models library in R (nlme), we evaluated all available relapse n = 10 and remission samples n = 40 from MOG-AAD patients and compared them with PHC n = 44 samples (Supplementary Table 1a and 1b). We found a significant reduction in TNFAIP3 serum levels in relapse samples as compared to remission samples (*P* = 0.04). There was also significant reduction in TNFAIP3 serum levels in relapse samples as compared to PHC (*P* = 0.0001). This indicated that low levels of TNFAIP3 are associated with the onset and subsequent relapses in MOG-AAD patients whereas patients at remission and healthy controls show high levels of TNFAIP3.

In order to assess if there were other proteins associated with relapse in MOG-AAD, we evaluated serum samples of 49 MOG-AAD patients (relapse samples n = 10 and remission samples n = 38) using a multiplex inflammatory panel made up of 92 proteins. Data was presented as normalized protein expression (NPX) values. After adjusting for age, sex and treatments, we found 7 biomarkers: FLT3 (Fms Related Tyrosine Kinase 3 Ligand, *P* = 0.0061), CDCP1 (CUB Domain Containing Protein 1, *P* = 0.008), IL12B (Interleukin 12B, *P* = 0.0284), NTF3 (Neurotrophin 3, *P* = 0.036), EIF4EBP1 (Eukaryotic Translation Initiation Factor 4E Binding Protein 1, *P* = 0.04), KITLG (KIT Ligand, *P* = 0.042), CCL2 (C–C Motif Chemokine Ligand 2, *P* = 0.046) and CCL25 (C–C Motif Chemokine Ligand 25, *P* = 0.05) that were significantly down regulated in relapse samples. However, these were no longer significant when corrected for multiple comparisons.

## Discussion

In this study we evaluated PBMC and serum samples from MOG-AAD patients at relapse and remission time points. We assessed for biomarkers by transcriptomic analysis. Utilizing 3 independent gene expression platforms, we identified TNFAIP3 as a relapse biomarker in MOG-AAD patients. Further we showed that TNFAIP3 protein level is reduced at relapse time point and increased at remission time point in MOG-AAD patient as well as in healthy controls. This was later confirmed in serum samples of MOG-AAD patients and healthy controls, where we found TNFAIP3 serum levels were decreased at relapse and increased at remission and in healthy controls.

MOG-AAD is a unique autoimmune demyelinating disease with a strong antigen-specific central memory Th1 and Th17 T cell response, and associated antibody production. TNFAIP3 gene, encoding the A20 protein, is emerging as a pivotal checkpoint in autoimmune diseases such as MS, rheumatoid arthritis and Crohn’s disease^38^. We found through transcriptomic analysis of central memory T cells (CMC) that TNFAIP3 gene expression is decreased during a relapse, and that this decrease correlates with a subsequent activation of NFκβ signaling. Protein analysis demonstrates that MOG-AAD relapse and remission samples respond differently to antigen stimulation, suggesting a dysregulation of TNAIP3 response, which may be dependent on cell state. We found that TNFAIP3 serum level decrements are associated with the onset of a relapse in individual MOG-AAD patients and thus has significant potential as a biomarker and therapeutic target for MOG-AAD and other autoimmune diseases.

The human TNFAIP3 gene is located on chromosome 6, and is also known as A20. It encodes the 790 amino acid protein, A20 that is, made of an N-terminal protease domain and seven Cys2-Cys2 zinc finger C-terminal domains. A20 is an ubiquitin-editing enzyme belonging to the ovarian tumor (OTU) proteases family of deubiquitinating (DUB) enzymes^23^. A20 functions as an E3 ubiquitin ligase as a result of its fourth zinc finger motif in the C-terminal domain TNFAIP3 is a key regulator of cellular processes including NFκβ activation and apoptosis^24–26^. TNFAIP3 suppresses cellular processes by down regulating NFκβ activation in part through DUB activity, ubiquitin-binding activity, and/or E3 ligase activity of critical signaling components including RIP1, TRAF6 and NEMO, upstream of the IKK complex^27–29,34^. It also binds directly to the C-terminus of IL-17RA^30^, and can decrease IL-17 responses through inhibition of p38^31^. Several stimuli, including TNFα, LPS, TLR and IL-1 through activation of NFκβ via the non-canonical pathway, increase TNFAIP3 mRNA expression^25,32^, thus initiating a negative feedback loop to regulate NFκβ^33^ via TRAF1/TRAF2^34^. TNFAIP3-deficient cells fail to terminate TNF-α-induced NFκβ responses^25^. Mice deficient in TNFAIP3 develop severe cachexia and inflammation in the liver, kidneys, intestines, joints, and bone marrow, and die prematurely^25^. Astrocytic expression of TNFAIP3 in EAE protects from CNS immune-mediated demyelination through suppression of chemokines^35^. Deletion of TNFAIP3 in microglia increases microglial cell number and affects microglial regulation of neuronal synaptic function and worsens demyelination in EAE through hyperactivation of the Nlrp3 inflammasome^36^. Thus loss of TNFAIP3 function and/or polymorphisms in the TNFAIP3 gene encoding for A20 protein is related to reduced A20 expression which thereby causes immune mediated inflammation and autoimmune diseases in humans^36^.

Activation of NFκβ through the canonical pathway related to TCR activation requires MALT1 signaling, which is suppressed by TNFAIP3 through deubiquitination^37^. In turn, MALT1 mediates rapid proteolytic cleavage and inactivation of TNFAIP3 after TCR stimulation, thus fine-tuning TCR activation^21^. MALT1 is also required for BCR activation and TNFAIP3 deletion is frequently observed in B cell lymphomas^38^.

Our results demonstrate that CD4+ T cell and serum decrements of TNFAIP3 are associated with clinical relapses in MOG-AAD patients and also associated with relative increases in NF-κβ expression, which is consistent with the role of TNFAIP3 in modulating NF-κβ activation. However these results could be due to the effects of strong antigen-specific activation of T cells in MHC-matched patients, or because of aberrant regulation of TNFAIP3 in this condition. Indeed, genetic polymorphisms of the TNFAIP3 gene have been described in several autoimmune diseases including rheumatoid arthritis, psoriasis, type 1 diabetes, inflammatory bowel disease, systemic lupus erythematosus (SLE), coronary artery disease and celiac disease^39–44^. Several TNFAIP3 intergenic polymorphisms have also been associated with MS susceptibility^45^. MOG-AAD is closely related to MS, however it has distinct clinical and radiological features. A comprehensive large-scale genomic analysis of MOG-AAD has not been reported thus far.

MOG-AAD, which is largely a pediatric disease, is highly sensitive to glucocorticoid treatment^7^. Glucocorticoids bind to the glucocorticoid receptor (GR) leading to immune suppression. TNFAIP3, which is an anti-inflammatory target of TNF-α, inhibitor of NF-κβ, is regulated by steroids including estrogen^46^ and glucocorticoids^47^. While stimulation with MOG antigen failed to induce TNFAIP3, dexamethasone stimulation, a potent glucocorticoid, partially rescued the expression of TNFAIP3 protein and transcriptome levels in the relapse sample thereby suggesting that the induction of TNFAIP3 by glucocorticoids can improve relapses in MOG-AAD patients.

Serum TNFAIP3 levels may be related to secretion by immune cells. The decrement in TNFAIP3 may be associated with antigen-stimulation of the TCR, thus associated with activation of antigen-specific T cells. In this study, we analyzed gene expression only in CMC T cells, which are integral in initiating antigen-specific immune response. However, B cells, monocytes and other cells also express TNFAIP3, and may be involved in eliciting TNFAIP3 serum levels. It is possible that antigen-specific responses in other diseases may be associated with similar findings. The serum level of TNFAIP3 varies between individual patients, and only decrements observed in longitudinal samples, but not absolute values were associated with relapses.

The utility of TNFAIP3 as a biomarker for relapses will need to be validated in additional MOG-AAD patients with age and gender-normalized comparisons in healthy controls. Here we demonstrate that decreased TNFAIP3 levels are associated with relapses in MOG-AAD patients. TNFAIP3 may not only indicate disease severity but also serve as a therapeutic target and a prognostic biomarker in MOG-AAD patients. Thus, further studies are required to evaluate the effect of increased expression of TNFAIP3 in patients with MOG-AAD and other related diseases.

Strengths of this study include carefully phenotyped cohorts of pediatric patients with MOG-AAD, and detailed cellular, transcriptomic and proteomic analysis. Limitations are the small number of subjects investigated, which reflects the challenges of obtaining multiple biological samples in pediatric patients. Steps are underway to further validate these results in separate cohorts of subjects, and to develop a set of multivariate proteomic and/or transcriptomic biomarkers for potential use in the clinical setting.

## Materials and methods

### Subjects and blood samples

All methods in this study were carried out in accordance with relevant guidelines and regulations. Subjects were selected from an ongoing biomarker study at the Partners Pediatric MS Center at Massachusetts General Hospital, which is approved by Partners Human Research Committee/Institutional Review Board for the use of human material. Parents of children signed an informed consent form. Peripheral venous blood was collected in lithium heparin blood collection tubes (Becton Dickinson, NJ, USA) from subjects after obtaining informed consent. We included 20 pediatric MOG-AAD patients and 44 age and sex-matched pediatric healthy controls (PHCs). MOG antibody testing was performed at the sample collection site by cell-based assay^6^, or at the Mayo Clinic as part of clinical care. The 20 MOG-AAD patients had the following diagnoses at the time of sample collection according to International Pediatric MS Study Group diagnostic criteria: 7 with MS, 7 with ADEM-ON, 1 with multiphasic ADEM, 1 with ADEM-TM, 1 with CIS, 1 with a demyelinating neurological disorder and 2 with NMO-SD^48^. Patients were diagnosed with NMO-SD if presenting with ON, TM and at least two of these 3 criteria: MRI evidence of a continuous spinal cord lesion, brain MRI that was non-diagnostic of MS, and NMO IgG seroposivity^48,49^. Out of the 20 MOG-AAD patients, 15 patients had longitudinal samples, including treated/untreated and relapse/non-relapse samples. Untreated samples were defined as no steroids or intravenous immunoglobulin nor disease modifying therapies within 30 days prior to sample collection. Samples within 30 days of a clinical relapse with new MRI lesions were defined as “relapse” samples or within 64 days of ongoing relapse symptoms, while samples at a non-relapse time point were defined as remission samples. One sample (#1.3) was identified as a pre-relapse sample, since the patient reported new mild clinical symptoms, but no radiological correlation was found (Table 1 and Supplementary Table 1 and b). A second sample (#5.5) was also termed pre-relapse since the patient had significant new headache not responsive to standard medication. Neurologists specialized in pediatric demyelinating disorders validated all clinical and radiological data (TC and GG).

### Cell stimulation assay and FACS analysis

Peripheral blood mononuclear cells (PBMCs) were isolated by Ficoll-Paque Plus (GE healthcare Biosciences AB, Sweden) density gradient centrifugation. Cells were cultured using Hybridomas and Lymphoid cells (HL-1, Lonza, MD, USA) media containing 5% of human serum (Valley Biomedical INC, VA, USA), L-Glutamine (Fisher Scientific, NH, USA), Penicillin–Streptomycin (Fisher Scientific, NH, USA), HEPES, non-essential amino acids (NEAA) and sodium pyruvate (all 3 from Lonza, MD, USA). PBMCs from 7 MOG-AAD patients of which 3 patients had longitudinal samples and 7 age and sex matched PHCs (Table 1) were plated at a density of 500,000 cells/well in a 96-well plate (Corning, ME, USA). Cells were stimulated by either one of the following 6 peptide conditions, (1) MOG p1-20, (2) MOG p35-55 (Immune Tolerance Network, USA), (3) MOG p119-130 (4) MOG p181-195 (5) MOG p186-200 (Genemed INC, CA, USA), (6) cocktail of myelin peptides consisting of Proteolipid Protein (PLP) 139–154, Myelin Basic Protein (MBP) 13–32, MBP 111–129 and MBP 146–170 (Immune Tolerance Network, USA) at 10 μg/ml. Cells were cultured for 7 days at 37 °C, 5% CO_2_ and 90% humidity.

To evaluate cytokine production, cells were further stimulated with Phorbol 12-myristate 13-acetate (PMA) at 33 ng/ml, Ionomycin at 166 ng/ml (Sigma Aldrich, MO, USA) and Golgi stop (BD Bioscience, CA, USA) for 4 h. After incubation cells were stained with the following antibodies, human anti-CD4 APC (RPA-T4, Biolegend Inc, CA, USA), anti-CD45RA-AF700 (HI100, Biolegend Inc, CA, USA), anti-CCR7-PE (GO43H7, Biolegend Inc, CA, USA) and live/dead fixable violet dead cell stain kit (Thermo Fischer Scientific, USA). Cells were then fixed and permeabilized with BD fixation and permeabilization buffer (BD Biosciences, CA, USA) and stained with the following intracellular cytokine antibodies: anti-IL17-PeCy7 (BL168, Biolegend Inc, CA, USA) and anti- IFNγ-PerCp (4S.B3, Biolegend Inc, CA, USA). Flow cytometry was performed on BD LSR II (BD Biosciences, CA, USA); data was analyzed using Flowjo vX.0.7 and the graphs were made using GraphPadPrism version 8.4.2 (464).

Live CD4+ T cells were selected from all lymphocytes and analyzed for expression of central memory cells (CMCs: CCR7+CD45RA−), effector memory cells (EMCs: CCR7−CD45RA−), and effector cells (CD45RA+). Intracellular pro-inflammatory cytokines, IL17+, IFNγ+, and IL17+IFNγ+ (double positive) were gated within CMCs and EMCs.

### Gene expression assays and data analysis

#### Single cell RNA sequencing (inDrop)

Single cell RNA sequencing was conducted in CMCs from CD4+ T cells isolated from MOG-AAD patient#1 with 3 untreated longitudinal samples, (1) at remission (MOG-AAD#1.2) (2) at a pre-relapse (MOG-AAD#1.3), two months prior to a relapse in which the patient experienced headache and mild visual symptoms with no radiological correlate and (3) at a relapse (MOG-AAD#1.4) characterized by bilateral weakness and multiple new MRI lesions (Table 1) following the inDrop technique as previously described^20,21^. InDrop single cell library sequencing was performed at the Single Cell Core (SCC), laboratory of Dr. Allon Klein, Department of Systems Biology at Harvard Medical School (HMS). The relapse sample failed sequencing, however the remission and pre-relapse samples were evaluable. For simplicity, moving forward we reassigned the pre-relapse sample to be a relapse sample.

Whole PBMCs were stimulated with anti-CD3 (OKT3, Ebioscience, CA, USA) and anti-CD28 (CD28.2, Ebioscience, CA, USA) at 0.5 μg/ml for 3 days. Cells were stained with human anti-CD4 APC (RPA-T4, Biolegend Inc, CA, USA), anti-CD45RA-AF700 (HI100, Biolegend Inc, CA, USA), anti-CCR7-PE (GO43H7, Biolegend Inc, CA, USA) and live/dead fixable violet dead cell stain kit (Thermo Fischer Scientific, USA). CMCs (CCR7+CD45RA−) were sorted from CD4+ T cells using BD FACS ARIA (BD Biosciences, CA, USA). 3,000 cells from a cell suspension comprising of CMCs from the 2 samples were isolated into droplets that contained lysis buffer. cDNA libraries were sequenced using the Illumina NextSeq 500 platform and analyzed following V3 Indrop criteria. After sequencing the raw BCL files were manually demultiplexed using the bcl2fastq (https://support.illumina.com/sequencing/sequencing_software/bcl2fastq-conversion-software.html) software by illumina. Reads obtained from bcl2fastq were further processed using the single-cell RNA-seq pipeline of the bcbio-nextgen (https://bcbio-nextgen.readthedocs.io/en/latest/contents/pipelines.html#single-cell-rna-seq) software suite. The single-cell RNA-seq pipeline inspected each read, performed alignment using RapMap^50^ and produced transcript level count matrix. This matrix was further processed with Seurat (https://www.biorxiv.org/content/early/2018/11/02/460147) where QC, filtering, log normalization and scaling was performed. The scaled data was further clustered using Seurat and visualized using TSNE^51^.

The TSNE plot was labeled with sample state (Relapse/Remission) to identify cluster differences between states. Since the clusters showed homogeneity between relapse and remission sample states, gene specific differences between the samples were assessed. “AverageExpression” function in Seurat was used to calculate average gene expression for the relapse and remission samples separately. The difference in the gene expression was calculated by subtracting the expression values from relapse and remission samples for each gene. The obtained list was further sorted to identify genes that were up regulated in relapse and remission samples respectively. Based on the difference in the gene expression, the top 5 genes were further evaluated.

#### Digital gene expression (DGE) sequencing

DGE sequencing was conducted in CMCs from CD4+ T cells isolated from the same MOG-AAD patient#1 with 3 untreated longitudinal samples at remission (MOG-AAD#1.2), pre-relapse (MOG-AAD#1.3) and relapse (MOG-AAD#1.4) as previously described (Table 1)^24–26^. PBMCs were stimulated with MOG peptides, p35-55, p119-130 and p181-195 at 10 μg/ml for 7 days. Cells were stained with human anti-CD4 APC (RPA-T4, Biolegend Inc, CA, USA), anti-CD45RA-AF700 (HI100, Biolegend Inc, CA, USA), anti-CCR7-PE (GO43H7, Biolegend Inc, CA, USA) and live/dead fixable violet dead cell stain kit (Thermo Fischer Scientific, USA). CMCs (CCR7+CD45RA−) were sorted from CD4+ T cells using BD FACS ARIA. Total RNA was isolated using Total RNA purification kit following manufacturer`s guidelines (Norgen, MA, USA). Based on the guidelines provided by the Broad institute for library preparation, RNA concentration for all samples was normalized to 5 ng/μl l in 10 μl of nuclease free water. Library sequencing was performed at The Broad Genomics Platform.

Raw BCL files generated through sequencing were further de-multiplexed using Picard (https://github.com/broadinstitute/picard) and the resulting FASTQ files where aligned to the human reference genome (GRCh38) using the STAR v2.4.2a^52^ aligner. Further QC was done using the RNA-seQC^53^ and transcript counts were produced using feature Counts function of the Subread package^54^. Before running the analysis, genes with low overall expression were removed from the analysis and the data was normalized using the DESeq2 package^55^. The graphs were made using GraphPadPrism version 8.4.2 (464).

#### Nanostring ncounter gene expression assay

Cell subsets were isolated from MOG-AAD patient#2 with 3 longitudinal samples, 1 at relapse (MOG-AAD#2.1) and 2 mycophenolate mofetil treated samples at remission (MOG-AAD#2.2 and MOG-AAD#2.3) (Table 1) were evaluated by NanoString array as previously described^31^. RNA expression of 770 genes was detected by nCounter XT Code-Set Gene Expression Assay, Human Autoimmune kit. CD4+ T cells, CD19+ B cells and CD14+monocytes were positively selected using micro beads and magnetically isolated using MACSQuant columns placed in the magnetic field of a MACS separator (Miltenyi Biotec, CA, USA). In order to achieve maximum purity, staining antibodies for anti-CD4-PE (OKT4, Biolegend Inc, CA, USA) anti-CD19-PE (HIB19, Biolegend Inc, CA, USA) and anti-CD14-FITC (M5E2, BD Biosciences, CA, USA) were added during magnetic separation. 7-AAD viability staining solution was used to separate the live cells from the dead. Further CD4+, CD19+ and CD14+ cells were sorted using BD FACS ARIA (BD Biosciences, CA, USA). Total RNA was isolated using Total RNA purification kit following manufacturer`s guidelines (Norgen, MA, USA). RNA concentration for all samples was normalized to 30 ng/μl l in 5 μl of nuclease free water. Hybridization protocol for nCounter XT Code-Set Gene Expression Assay was performed following the manufacturer`s instructions. Data were normalized and analyzed using nSolver software via the geometric mean of included housekeeping genes. The graphs were made using GraphPadPrism version 8.4.2 (464).

#### Quantification by real time PCR

CD4+ T cells from 7 MOG-AAD patients (longitudinal n = 5/7, relapse n = 7, remission n = 8, total n = 15, Table 1) were isolated as previously described. Total RNA was isolated using Total RNA purification kit following manufacturer`s guidelines (Norgen, MA, USA). RNA concentration for all samples was determined using NanoDrop 2000/2000c Spectrophotometer (Thermo fischer scientific, USA). First-strand cDNA synthesis was performed for each RNA sample from 50 ng of total RNA using SuperScript IV VILO Master Mix (Thermo fischer scientific, USA) following manufacturer’s guidelines. qPCR was performed using FAM-labeled primers for TNFAIP3, Hs00234713_m1 and GAPDH, Hs99999905_m1 with TaqMan Fast Universal qPCR Master Mix (No ampErase Uracil *N*-Glycosylase, Thermo fischer scientific, USA). Samples were run in duplicates on QuantStudio 7 Flex (Applied Biosystems, Life Technologies, USA). GAPDH gene was used as an endogenous control to normalize for differences in the amount of total RNA in each sample, and all values are shown as relative expression. The graphs were made using GraphPadPrism version 8.4.2 (464).

### SDS-PAGE and western blot

PBMCs from an untreated MOG-AAD patient#3 with 2 longitudinal samples at relapse (MOG-AAD#3.1) and non-relapse (remission, MOG-AAD#3.2) (Table 1) were cultured at a density of 250,000 cells/well in a 96-well plate (Corning, ME, USA) for 4, 8, 16 and 24 h under the following 2 conditions: (1) MOG antigen at 1 μg/ml (peptide cocktail comprising of MOG p1-20, p35-55, p119-130, p181-195 and p186-200, Sigma Aldrich, MO, USA), and (2) MOG antigen at 1 μg/ml with Dexamethasone at 100 nM (Sigma Aldrich, MO, USA). PBMCs for an additional MOG-AAD patient#2 with 2 longitudinal samples, untreated at relapse (MOG-AAD#2.1) and treated with mycophenolate mofetil at non-relapse (remission, MOG-AAD#2.3) (Table 1) were cultured similarly under the following 5 conditions: (1) Exvivo (unstimulated) (2) MOG antigen stimulation at a lower dose of 1 μg/ml, (3) MOG antigen stimulation at a higher dose of 10 μg/ml (4) MOG antigen stimulation with dexamethasone at a lower dose of 1 μg + 100 nM, and (5) MOG antigen stimulation with dexamethasone at a higher dose of 10 μg + 1000 nM. Cells were lysed with RIPA lysis buffer (Thermo Fisher Scientific, MA, USA) supplemented with protease and phosphatase inhibitor cocktails (Thermo Fisher Scientific, MA, USA). Total protein was determined by Pierce BCA protein assay following manufacturer’s instructions (Thermo Fisher Scientific, MA, USA). Samples were prepared with 30 μg of cell lysate, loading buffer (Life Technologies, OR, USA), and reducing agent (Life Technologies, OR, USA) and then heated for 10 min at 95 °C before use. Samples were run on 4–12% Bis Tris gels (Invitrogen, CA, USA) and transferred to polyvinylidene difluoride membranes (PVDF) (Immobilon-P, Millipore Sigma, MA, USA). Membranes were blocked using 5% BSA (Sigma Aldrich, MO, USA) and blotted overnight at 4 °C with antibodies against anti-TNFAIP3 (ab92324, 1:1,000), anti- NFκβ p50 (ab32360, 1:2000) Abcam, MA, USA, anti-phospho NFκβ p65 (3033L, 1:2000), anti- NFκβ p65 (8242S, 1:2000), and anti- β-actin (4970S, 1:4,000) Cell signaling, MA, USA. Membranes were incubated with secondary antibody anti-rabbit IgG HRP linked (7074P2, 1:10,000, Cell signaling, MA, USA) for 1 h at 37 °C. Immunoblots were developed using KwikQuant western blot detection kit (Kindle biosciences, CT, USA). Protein bands were quantified using ImageJ version 1.53b and normalized to their respective β-actin. The graphs were made using GraphPadPrism version 8.4.2 (464).

### ELISA

TNFAIP3 concentration in serum samples of 50 MOG-AAD patients (Table 1 and Supplementary Table 1) with relapse samples n = 10, remission samples n = 40 and PHC n = 44 was assessed by commercially available TNFAIP3 ELISA kit (MyBiosource, CA, USA) following manufacturer’s instructions. All samples were tested in duplicates. Detection range of the assay was 23.5 pg/ml-1500 pg/ml. The Intra-assay precision CV was < 8% and Inter-assay precision CV was < 10%. The optical density was determined at 450 nm and the reference wavelength was set at 560 nm. Nonlinear Mixed-Effects Models library in R (nlme) was used for statistical analysis. The graphs were made using GraphPadPrism version 8.4.2 (464).

Multiplex panel comprising of 92 protein biomarkers (Olink Proteomics, Uppsala, Sweden) was used to assess serum samples of 49 MOG-AAD patients (Table 1 and Supplementary Table 1) with relapse samples n = 11 and remission samples n = 38 following manufacturer’s instructions. The Intra-assay precision CV was < 15% and Inter-assay precision CV was < 25%. Data from the analyzed protein biomarkers was presented in normalized protein expression (NPX) values, Olink Proteomics’s arbitrary unit on log2 scale.^56,57^.

### Disclosures

SS-none, HL-none, GG-none, RR-none, TR- none, TC has received personal compensation for advisory board/consulting for Biogen-Idec, Merck Serono, Novartis, Sanofi, Bayer, Celgene, Alexion and has received financial support for research activities from Merck Serono and Novartis Pharmaceuticals, the Department of Defense, National MS Society, the Guthy-Jackson Charitable Foundation and the Peabody Foundation.

## Supplementary information

Supplementary Legends.

Supplementary File1.
