# Supplementary material for: Identification of TNFAIP3 as relapse biomarker and potential therapeutic target for MOG antibody associated diseases
Source: Sci Rep. 2020 Jul 24;10:12405. doi: 10.1038/s41598-020-69182-w (PMC7381621; doi:10.1038/s41598-020-69182-w)
Supplement: Supplementary file 1 — Supplementary Legends. [file 41598_2020_69182_MOESM1_ESM.docx]

**SUPPLEMENTARY FIGURES:**

**Supplementary Figure 1: Genes up-regulated during remission and pre-relapse sample of an untreated MOG-AAD patient by single cell RNA sequencing:** Single cell RNA sequencing (inDrop) was performed on an untreated MOG-AAD patient#1 with 2 longitudinal samples, 1) Remission (MOG-AAD#1.1) and 2) Pre-relapse (MOG-AAD#1.2) as described in Materials and Methods. cDNA libraries were sequenced using the Illumina NextSeq 500 platform and analyzed following V3 Indrop criteria. After sequencing the raw BCL files were demultiplexed using bcl2fastq software by illumina (<https://support.illumina.com/sequencing/sequencing_software/bcl2fastq-conversion-software.html>). Reads obtained from bcl2fastq were further processed using the single-cell RNA-seq pipeline of the bcbio-nextgen (<https://bcbio-nextgen.readthedocs.io/en/latest/contents/pipelines.html#single-cell-rna-seq>) software suite. The scaled data was further clustered using Seurat and visualized using TSNE (<https://www.biorxiv.org/content/early/2018/11/02/460147>). **(A)** Differential expression of top 5 genes, HILPDA, MXI-1, BNIP3, TNFAIP3 and FAM162A, that was unregulated at remission. **(B)** Differential expression of top 5 genes, SNRPG, EWSR1, AL137058.2, HMGN1 and LRRC75A-AS1, that was unregulated at relapse.

**Supplementary Figure 2: Genes up-regulated during remission and relapse of an untreated MOG-AAD patient by Digital gene expression (DGE) sequencing:** Digital Gene Expression (DGE) sequencing was performed on an untreated MOG-AAD patient#1 with 3 longitudinal samples, 1) Rm (remission, MOG-AAD#1.1), 2) PR (pre-relapse, MOG-AAD#1.2), and 3) R (relapse, MOG-AAD#1.3) as described in Materials and Methods. The graphs were made using GraphPadPrism version 8.4.2 (464) **(A)** HILPDA, MXI-1, BNIP3 and FAM162A were up regulated during remission by DGE sequencing, same as single cell sequencing **(B)** SNRPG, EWSR1, HMGN1 and LRRC75A-AS1 did not follow a similar trend by DGE sequencing as it did with single cell sequencing. Gene expression for AL137058.2 was below the detection limit by DGE sequencing.

**Supplementary Figure 3:** PBMCs from a MOG-AAD patient#2 (untreated, relapse MOG-AAD#2.1 and treated with mycophenolate mofetil, non-relapse MOG-AAD#2.3) were cultured with 5 conditions, analyzed by SDS-PAGE and followed by western blot for TNFAIP3 at the indicated time points (hours) as described in Materials and Methods. **(A)** Western blot images for TNFAIP3 after 4 hours of stimulation **(B)** Western blot images for TNFAIP3 after 16 hours of stimulation **(C)** Western blot images for TNFAIP3 after 24 hours of stimulation **(D)** Dose response of TNFAIP3 in a relapse MOG-AAD patient#2.1 to MOG antigen stimulation (lower dose at 1μg/ml and higher dose at 10μg/ml) and MOG+dexamethasone stimulation (lower dose at 1μg+100nm and higher dose at 10μg+1000nm) at 4, 16 and 24 hours **(E)** Dose response of TNFAIP3 in a non-relapse MOG-AAD patient#2.3 to MOG antigen stimulation (lower dose at 1μg/ml and higher dose at 10μg/ml) and MOG+dexamethasone stimulation (lower dose at 1μg+100nm and higher dose at 10μg+1000nm) at 4, 16 and 24 hours **(F)** Western blot TNFAIP3 expression analysis in unstimulated (exvivo) PBMCs from a relapse MOG-AAD#2.1 and non-relapse MOG-AAD #2.3 patient at 4 and 24 hours. Protein bands were quantified using ImageJ version 1.53b and normalized to their respective β-actin. The graphs were made using GraphPadPrism version 8.4.2 (464). Full-length blots/gels for TNFAIP3 and β**-**Actin are presented in Supplementary Figure 6.

**Supplementary Figure 4:** PBMCs from a MOG-AAD patient#2 (untreated, relapse MOG-AAD#2.1 and treated with mycophenolate mofetil, non-relapse MOG-AAD#2.3) were stimulated with a lower dose of MOG antigen at 1μg/ml, analyzed by SDS-PAGE and followed by western blot for TNFAIP3 and NFκβ subunits p50 and p65 at the indicated time points (hours) as described in Materials and Methods. **(A)** Western blot images for TNFAIP3 and NFκβ subunits p50 and p65 in relapse and non-relapse MOG-AAD patient#2 **(B)** Correlation of TNFAIP3 expression with NFκβ subunit p50 expression in a relapse MOG-AAD patient#2.1 **(C)** Correlation of TNFAIP3 expression with NFκβ subunit p65 expression in a relapse MOG-AAD patient#2.1. Protein bands were quantified using ImageJ version 1.53b and normalized to their respective β-actin. The graphs were made using GraphPadPrism version 8.4.2 (464). Full-length blots/gels for TNFAIP3, NFκβ subunits p50 and phospho-p65/p65 and β**-**Actin are presented in Supplementary Figure 7.

**Supplementary Figure 5:** PBMCs from a MOG-AAD patient#3 at a relapse (MOG-AAD#3.1) and non-relapse (MOG-AAD#3.2) time point were cultured under different conditions, analyzed by SDS-PAGE and followed by western blot for TNFAIP3 at the indicated time points (hours) as described in Materials and Methods. **(A)** Full-length blots/gels for TNFAIP3 and β**-**Actin after MOG antigen stimulation at 1μg/ml (peptide cocktail comprising of MOG p1-20, p35-55, p119-130, p181-195 and p186-200). **(B)** Full-length blots/gels for TNFAIP3 and β**-**Actin after MOG antigen+dexamethasone stimulation at 1μg+100nm.

**Supplementary Figure 6:** PBMCs from a MOG-AAD patient#2 (untreated, relapse MOG-AAD#2.1 and treated with mycophenolate mofetil, non-relapse MOG-AAD#2.3) were cultured with 5 conditions, analyzed by SDS-PAGE and followed by western blot for TNFAIP3 at the indicated time points (hours) as described in Materials and Methods. **(A)** Full-length blots/gels for TNFAIP3 and β**-**Actin after 4 hours of stimulation **(B)** Full-length blots/gels for TNFAIP3 and β**-**Actin after 16 hours of stimulation **(C)** Full-length blots/gels for TNFAIP3 and β**-**Actin after 24 hours of stimulation.

**Supplementary Figure 7:** PBMCs from a MOG-AAD patient#2 (untreated, relapse MOG-AAD#2.1 and treated with mycophenolate mofetil, non-relapse MOG-AAD#2.3) were stimulated with a lower dose of MOG antigen at 1μg/ml, analyzed by SDS-PAGE and followed by western blot for TNFAIP3 and NFκβ subunits p50 and p65 at the indicated time points (hours) as described in Materials and Methods. **(A)** Full-length blots/gels for TNFAIP3 in relapse and non-relapse MOG-AAD patient#2 **(B)** Full-length blots/gels for NFκβ subunit p50 in relapse and non-relapse MOG-AAD patient#2 **(C)** Full-length blots/gels for NFκβ subunit phospho-p65 in relapse and non-relapse MOG-AAD patient#2. **(D)** Full-length blots/gels for NFκβ subunit p65 in relapse and non-relapse MOG-AAD patient#2. **(E)** Full-length blots/gels for β**-**Actin in relapse and non-relapse MOG-AAD patient#2.
