# Supplementary material for: Identification of TNFAIP3 as relapse biomarker and potential therapeutic target for MOG antibody associated diseases
Source: Sci Rep. 2020 Jul 24;10:12405. doi: 10.1038/s41598-020-69182-w (PMC7381621; doi:10.1038/s41598-020-69182-w)
Supplement: Supplementary file 2 — Supplementary File1. [file 41598_2020_69182_MOESM2_ESM.pptx]

## Slide 1
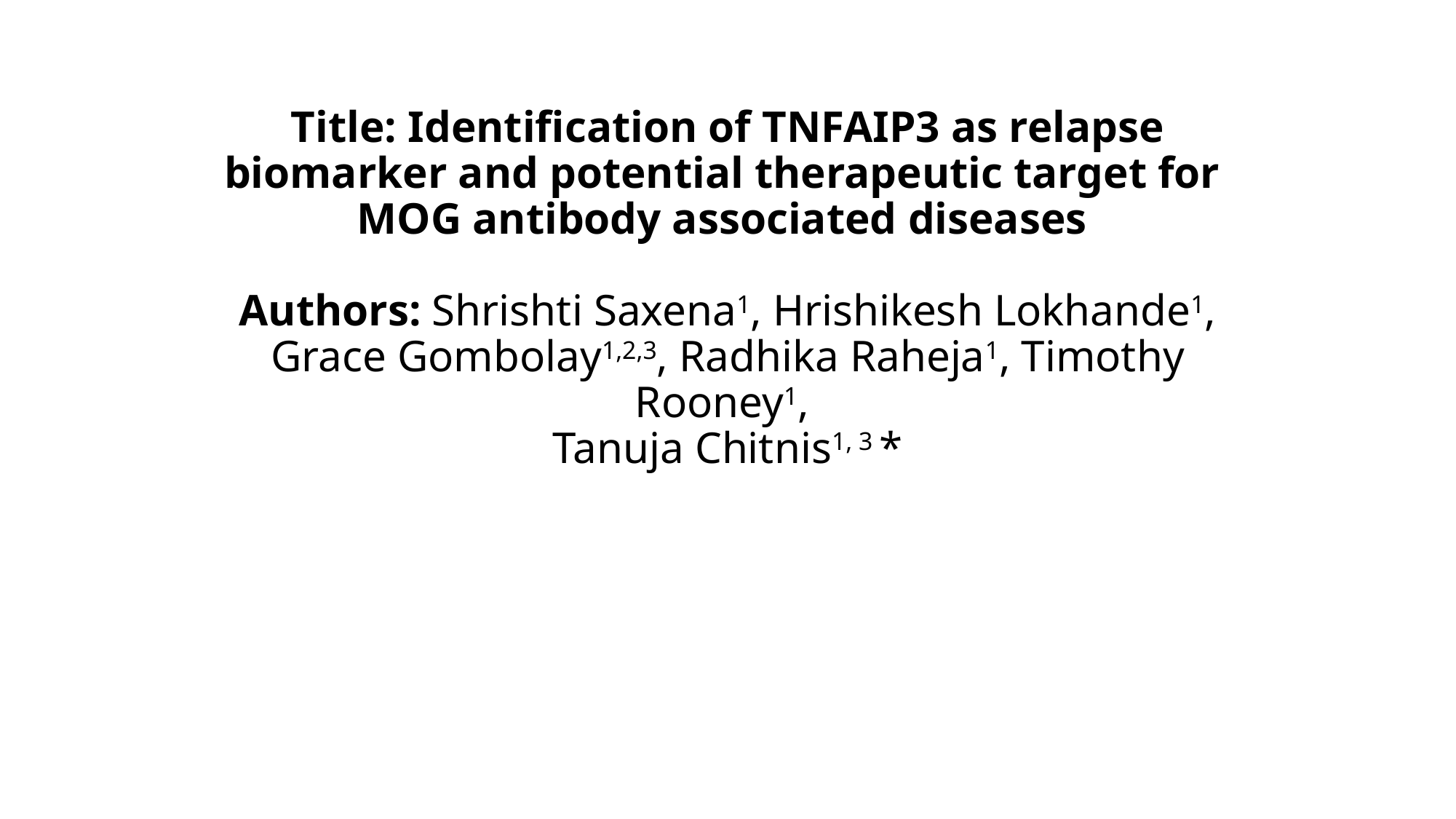

# Title: Identification of TNFAIP3 as relapse biomarker and potential therapeutic target for MOG antibody associated diseases Authors: Shrishti Saxena1, Hrishikesh Lokhande1, Grace Gombolay1,2,3, Radhika Raheja1, Timothy Rooney1, Tanuja Chitnis1, 3 *

## Slide 2
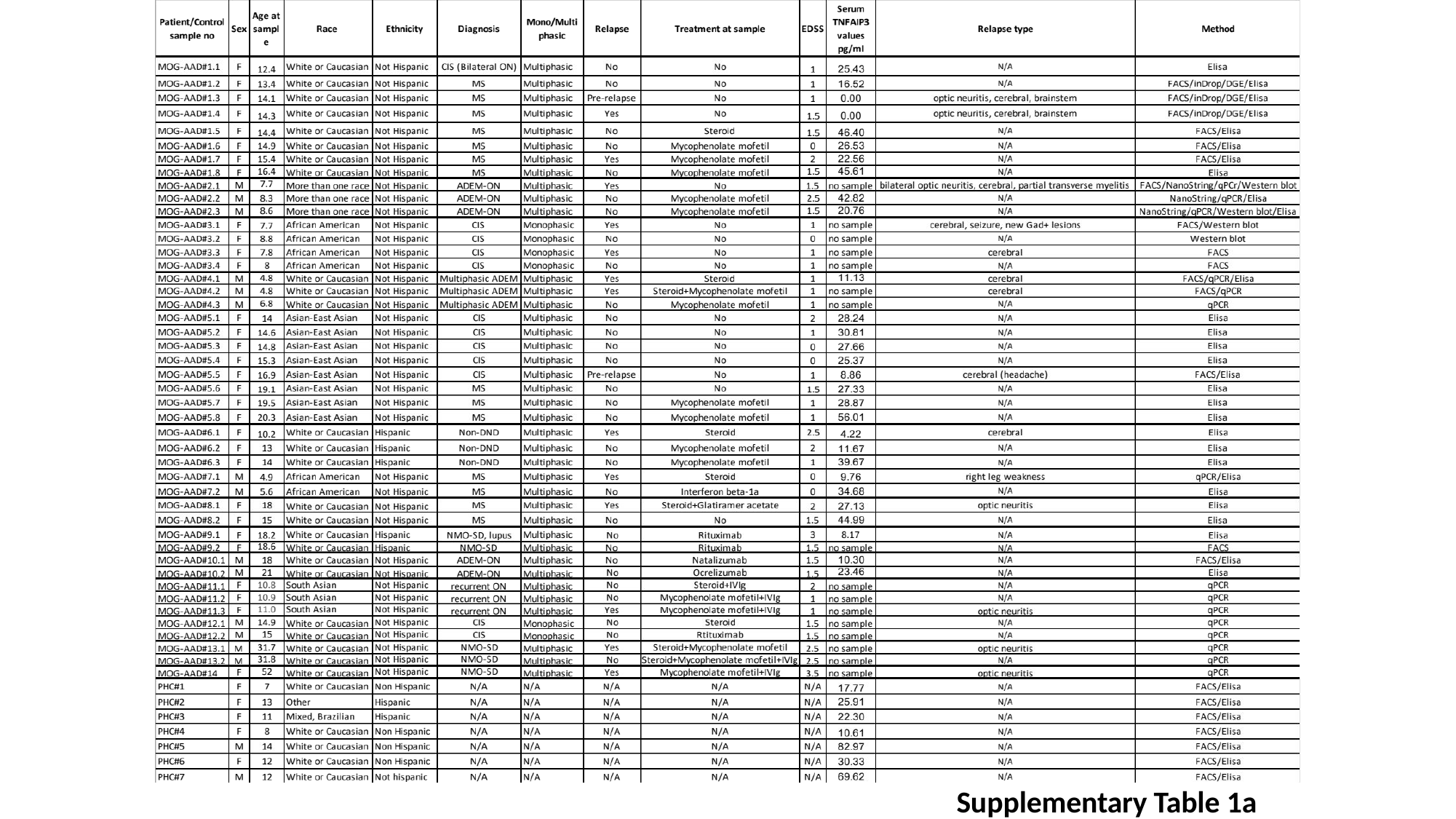

Supplementary Table 1a

## Slide 3
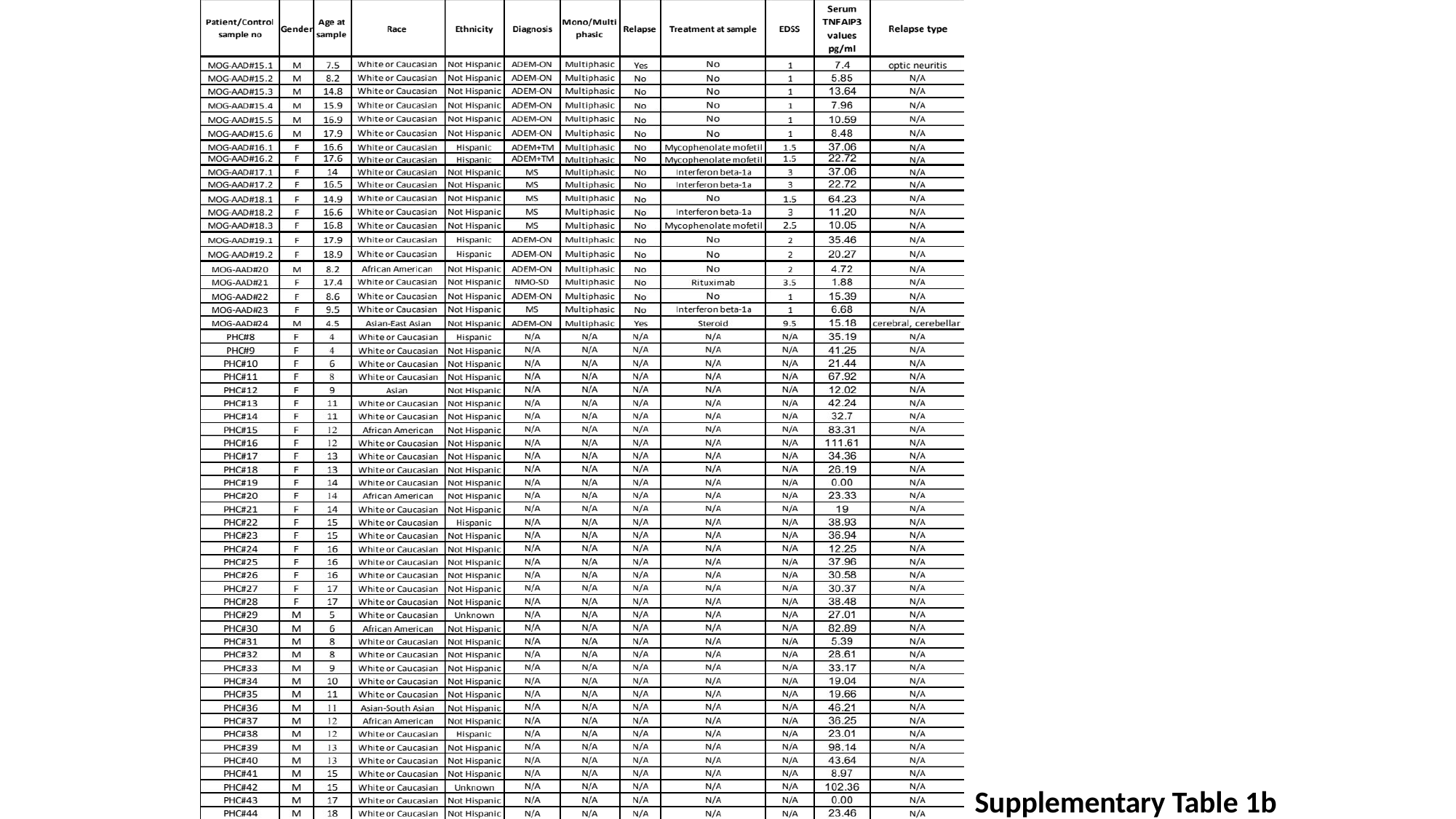

Supplementary Table 1b

## Slide 4
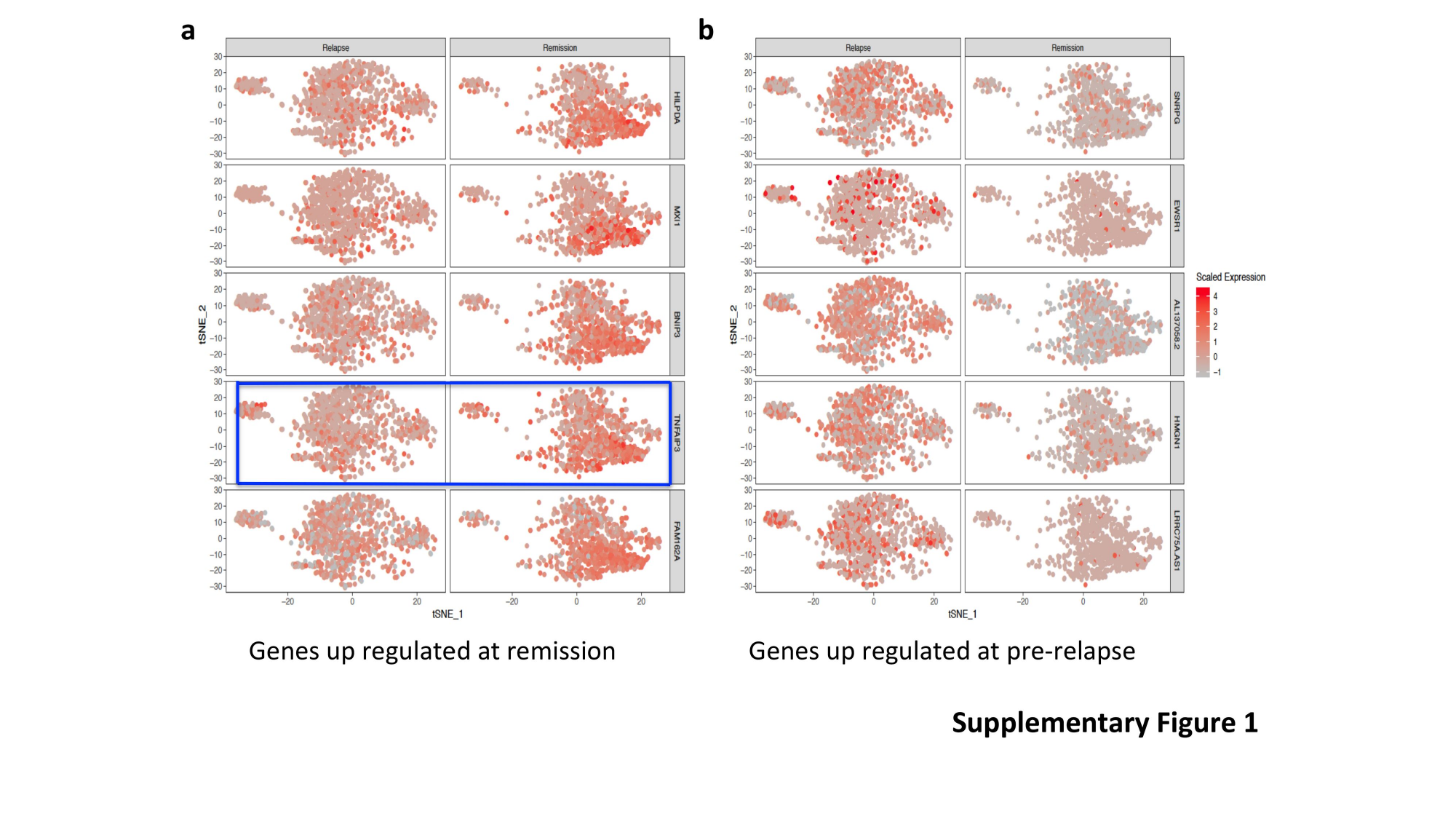

## Slide 5
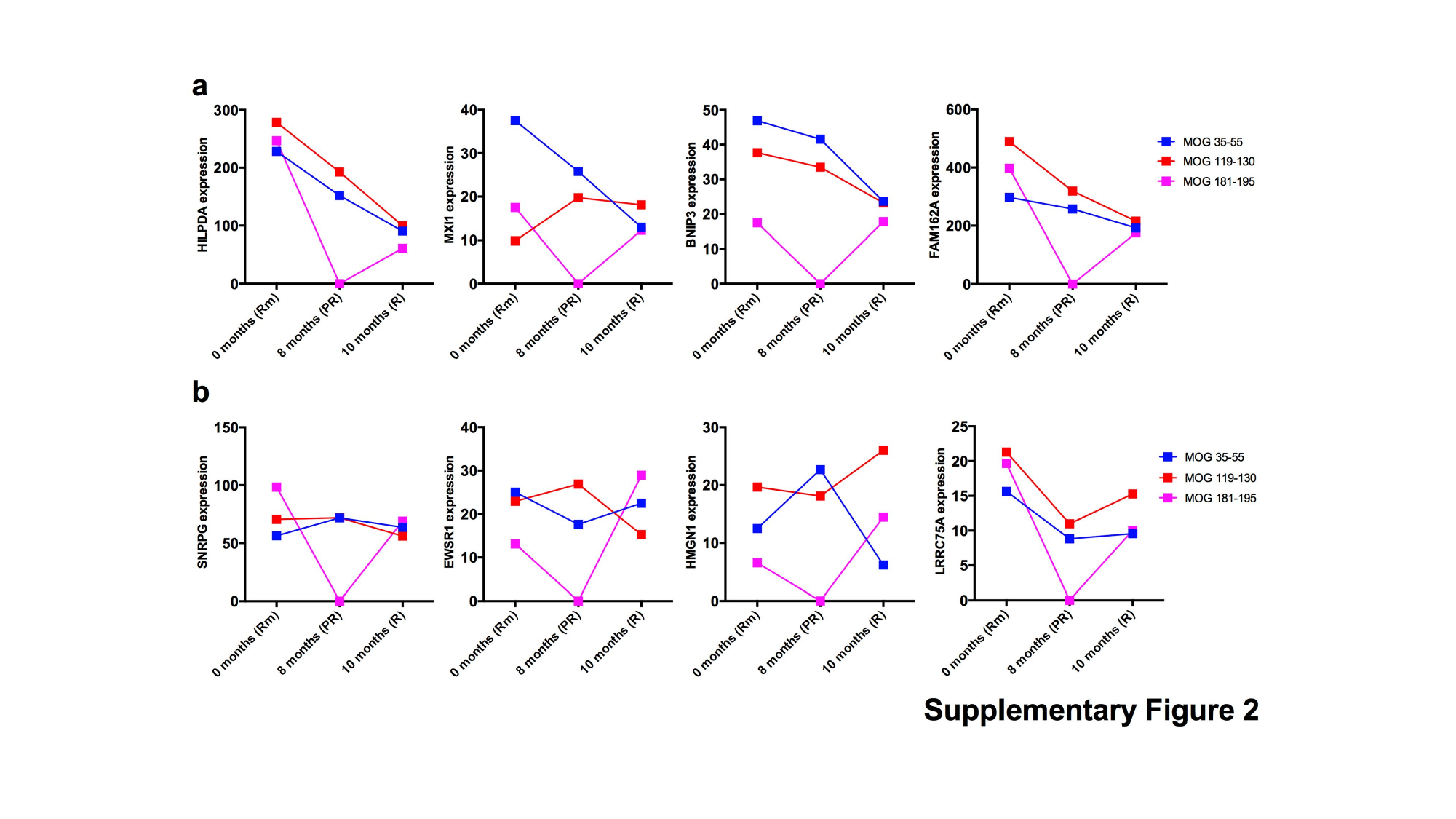

## Slide 6
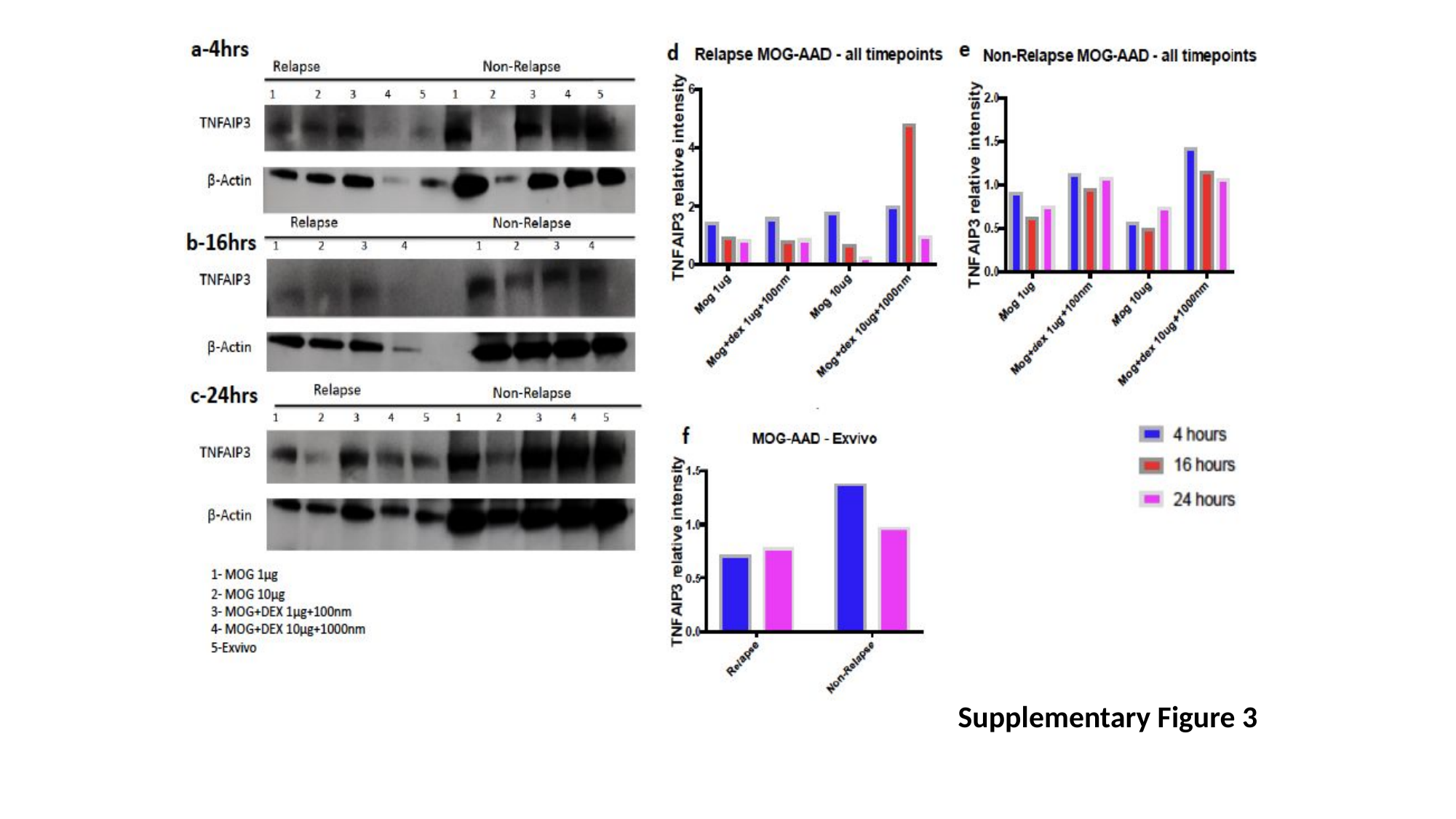

Supplementary Figure 3

## Slide 7
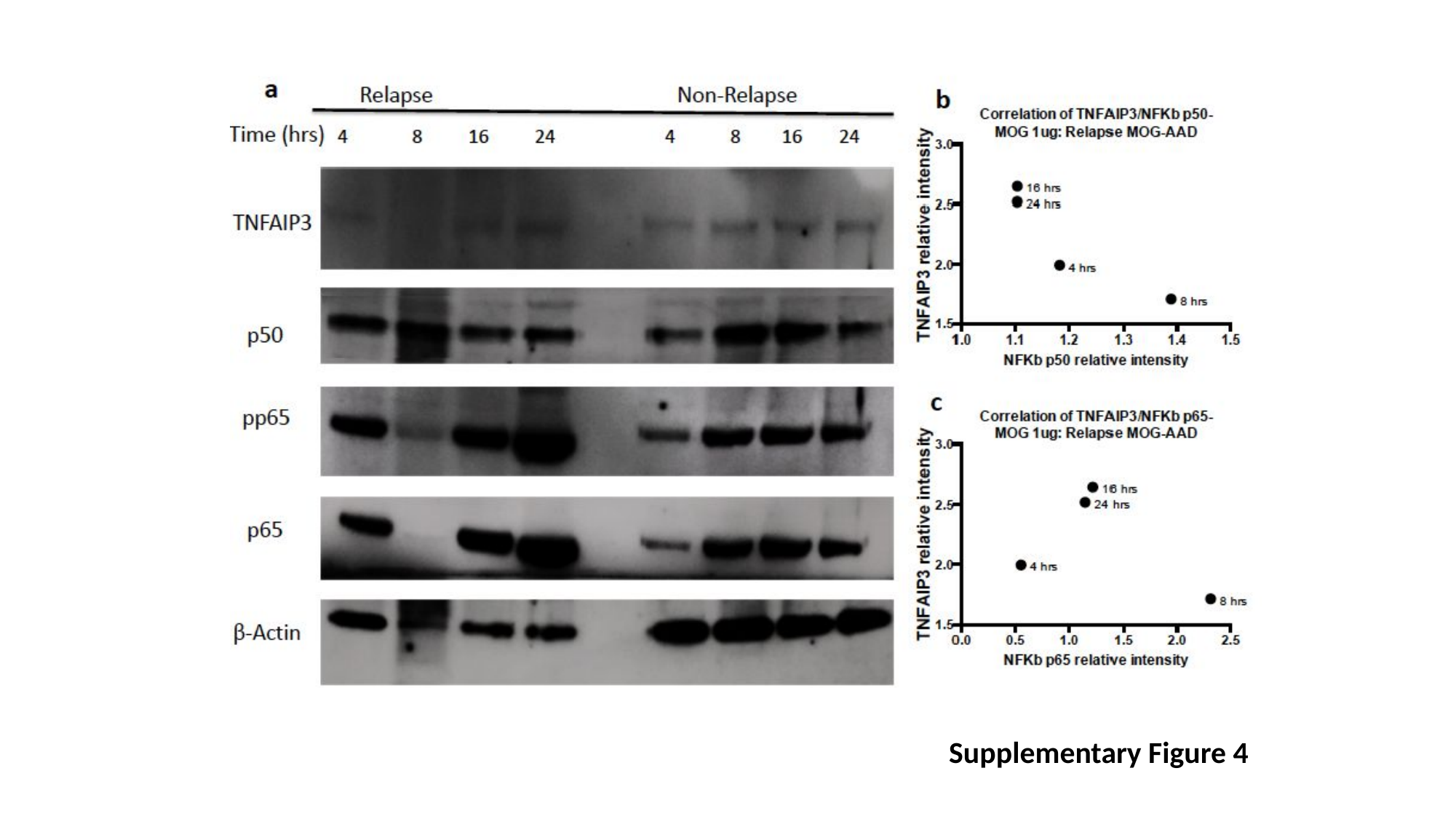

Supplementary Figure 4

## Slide 8
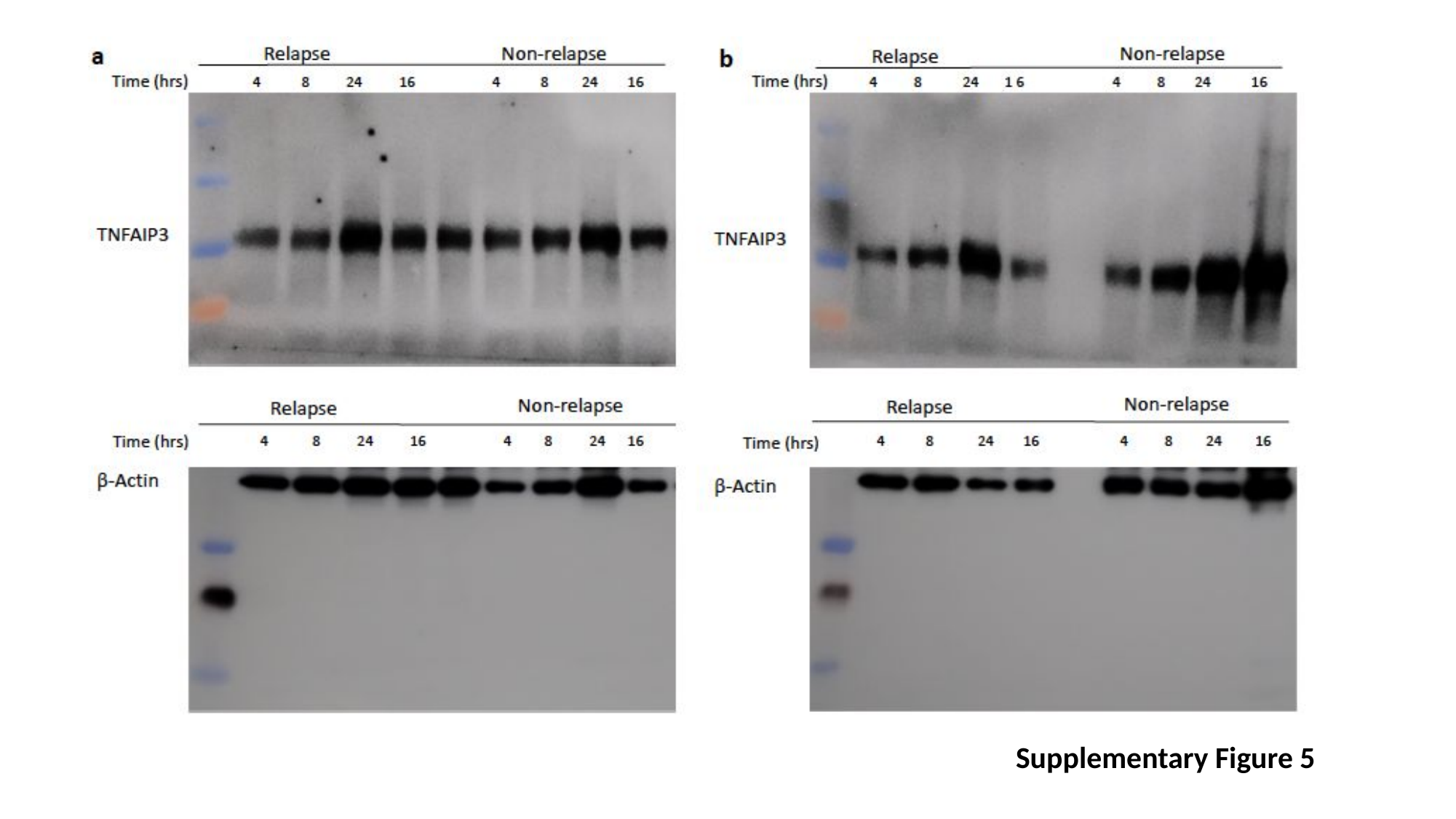

Supplementary Figure 5

## Slide 9
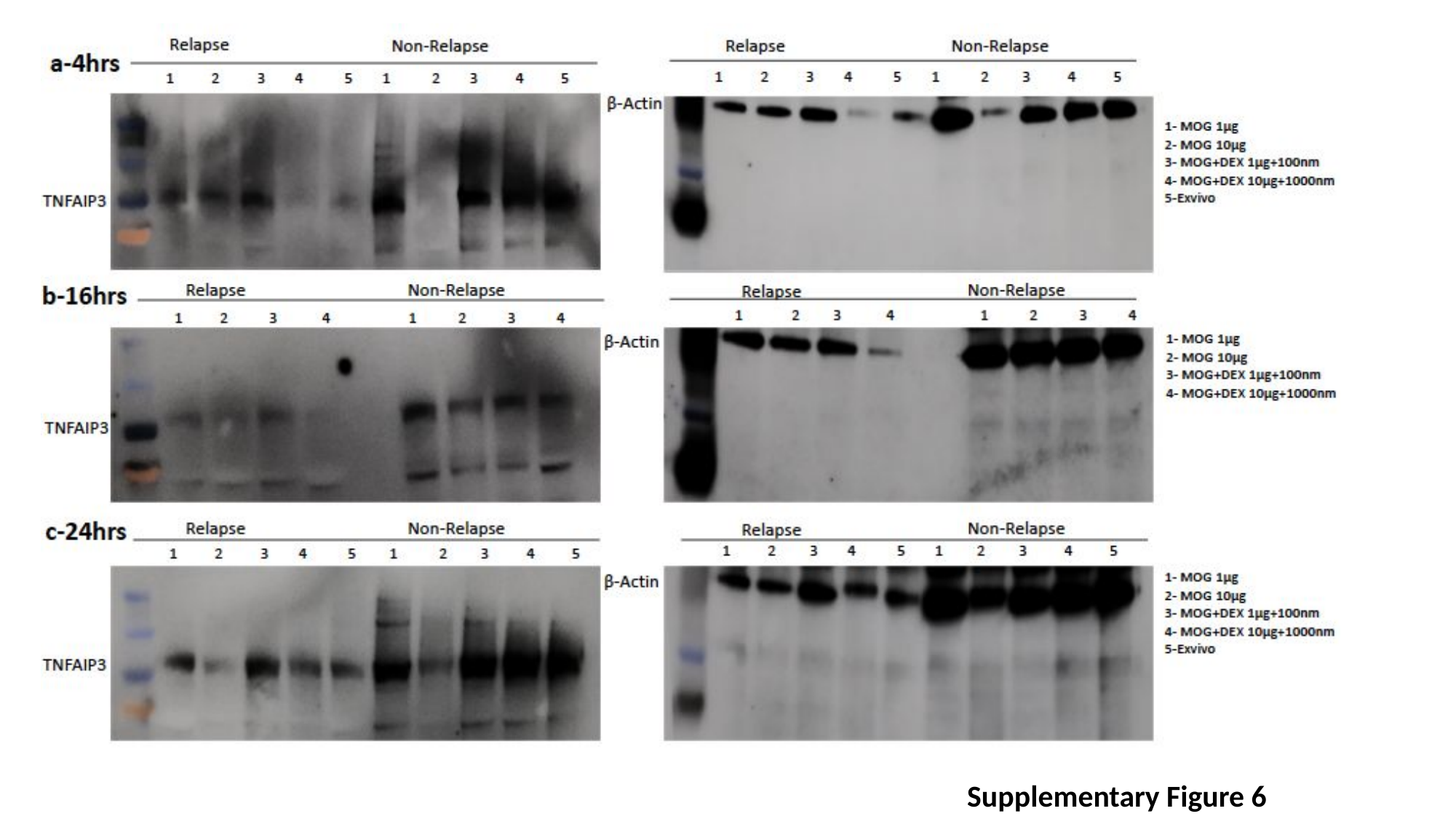

Supplementary Figure 6

## Slide 10
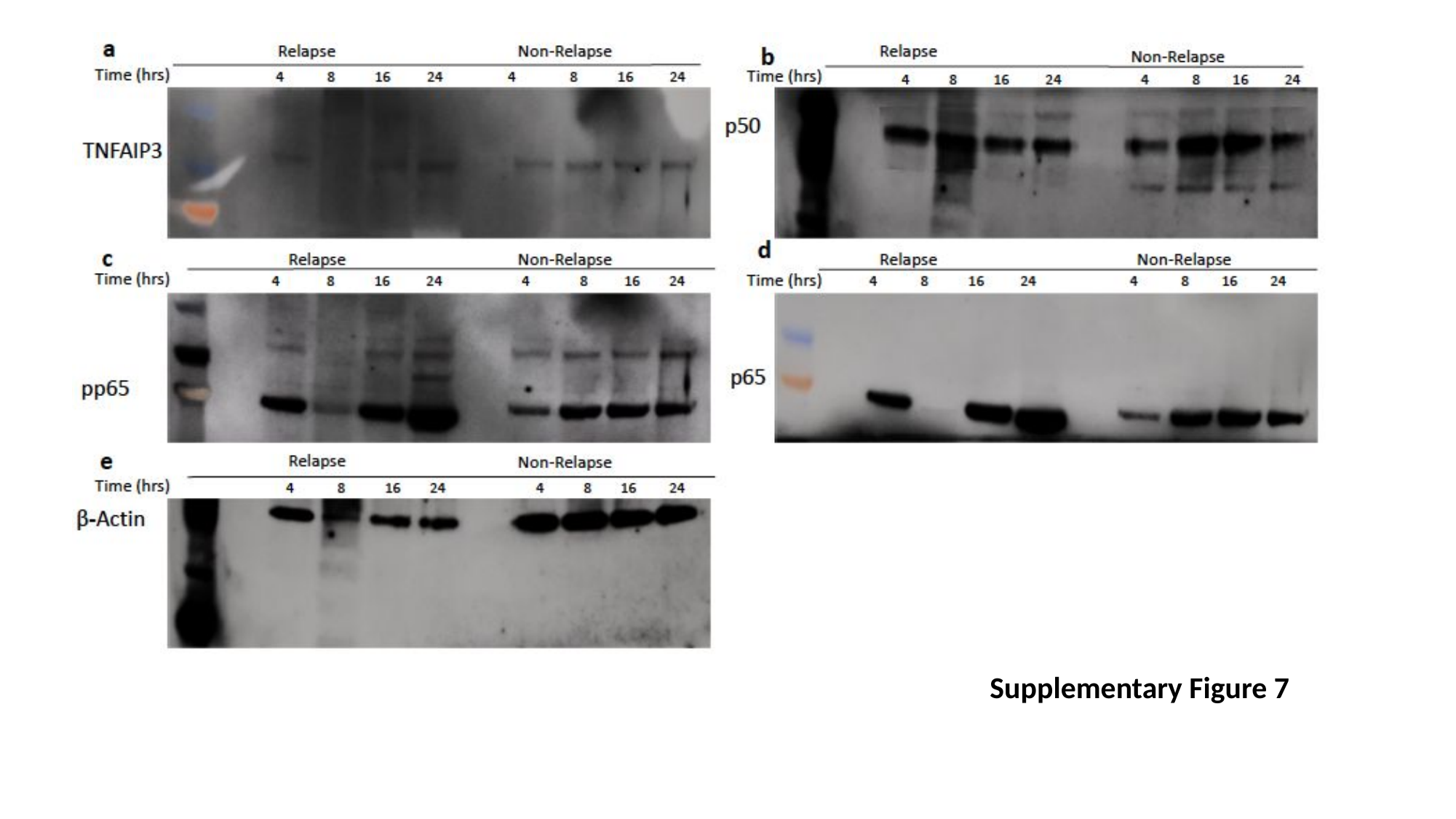

Supplementary Figure 7
